# Derivation of a fifteen gene prognostic panel for six cancers

**DOI:** 10.1038/srep13248

**Published:** 2015-08-14

**Authors:** Mamata F. Khirade, Girdhari Lal, Sharmila A. Bapat

**Affiliations:** 1 National Centre for Cell Science, NCCS Complex, Pune 411007, India

## Abstract

The hallmarks of cancer deem biological pathways and molecules to be conserved. This approach may be useful for deriving a prognostic gene signature. Weighted Gene Co-expression Network Analysis of gene expression datasets in eleven cancer types identified modules of highly correlated genes and interactive networks conserved across glioblastoma, breast, ovary, colon, rectal and lung cancers, from which a universal classifier for tumor stratification was extracted. Specific conserved gene modules were validated across different microarray platforms and datasets. Strikingly, preserved genes within these modules defined regulatory networks associated with immune regulation, cell differentiation, metastases, cell migration, metastases, oncogenic transformation, and resistance to apoptosis and senescence, with AIF1 and PRRX1 being suggested to be master regulators governing these biological processes. A universal classifier from these conserved networks enabled execution of common set of principles across different cancers that revealed distinct, differential correlation of biological functions with patient survival in a cancer-specific manner. Correlation analysis further identified a panel of 15 risk genes with potential prognostic value, termed as the GBOCRL-IIPr panel [(GBM-Breast-Ovary-Colon-Rectal-Lung)–Immune–Invasion–Prognosis], that surprisingly, were not amongst the master regulators or important network hubs. This panel may now be integrated in predicting patient outcomes in the six cancers.

Current trends in cancer management focusing on tumor heterogeneity emphasize a need for patient stratification into discrete molecular subtypes to achieve better disease management^1^,^2^,^3^. Information thus obtained can provide insights into understanding tumor progression, mechanisms of drug resistance and invasion, besides identifying novel therapeutic targets^4^. More importantly, patient care has been significantly enhanced as a result of biomarker based stratification of individual cancers like breast cancer, acute myeloid leukemia, high-grade gliomas, head and neck carcinomas and renal cell carcinoma^5^,^6^,^7^,^8^,^9^,^10^. The identification of cancer-specific gene signatures in disease prognosis and treatment also remains a robust approach towards personalized medicine. Unfortunately, such approaches can increase the demands on drug discovery to an unpractical level^11^,^12^. Some strategies to overcome this emerge from the realization that within the chaos generated by heterogeneous molecular profiles of tumor exist ordered transformation-associated pathways which are likely to be conserved across different cancers^13^. This has led to the identification of “biological signatures” that identify common functions from conserved gene networks and processes^14^. Some of the more popular signatures include stromal, invasive, stem cell, epithelial-mesenchymal transition (EMT), etc^15^,^16^,^17^,^18^,^19^, that are now proposed to be useful towards common drug target identification^20^. These signature components may be further applied for tumor classification across different tumor types to resolve similar sub-types across cancers wherein similar therapeutic regimes may be possible. This further raises the possibility that derivation of similar gene expression patterns and networks that drive transformation and tumor progression could in the long run improved disease management.

In an earlier approach, we had evaluated Weighted Gene Correlation Network Analysis (WGCNA) towards ovarian cancer tumor stratification and validated the suggested biological functions of associated gene networks^21^. In consequent informal analysis with other datasets, application of the same scheme of generating an adjacency matrix and organizing expression intensities for identification of functional modules of correlating genes^22^,^23^ suggestively generated modules that appeared to be similar across different tumor types. This led to the initiation of a defined study to probe the validity of this observation. Thus, eleven human tumor gene expression datasets from The Cancer Genome Atlas (TCGA) were thus examined. Our analysis affirmed the conservation of some modules and genes in a subset of six of these eleven cancers. We further studied the biological functions associated with these to identify master regulators that could possibly drive the specific phenotype. This also led to the extraction of a common classifier from the network components and development of similar stratification strategies across these cancers. Further probing the correlations with patient survival led to the identification of risk genes that support this classification and predict subtypes with similar features in different cancers. Finally, we derived a 15 signature gene panel which can be applied in the predicting prognosis for six cancers.

## Results

### Gene co-expression relationship and network analyses identified discrete modules across different cancers

**Figure 1:**
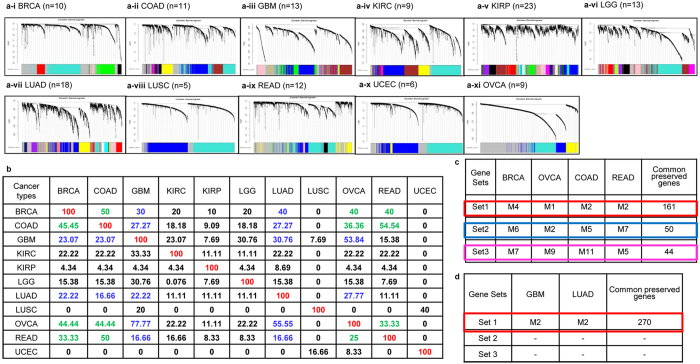
(**a**) Cluster dendrograms for (**a**-**i** to **a**-**xi**) BRCA, COAD, GBM, KIRC, KIRP, LGG, LUAD, LUSC, READ, UCEC, OVCA, each colour represents a specific module, n = number of modules; (**b**) Percentage module preservation across different cancers (computation details in methods)—numbers in diagonal denote module preservation within each cancer type, those in bold denote strong (across BRCA-COAD-OVCA-READ) and moderate module preservation (GBM-LUAD); (**c**) Strongly Preserved modules and gene sets BRCA-COAD-OVCA-READ; d. Moderately Preserved modules and gene sets in GBM-LUAD.

Statistically significant differentially expressed genes were identified in normalized gene expression data from the TCGA depository for eleven cancer types and individually subjected to Weighted Gene Correlation Network Analysis (WGCNA). For each cancer this achieved clustering into a specific number of gene groups referred to as modules (Supplementary Dataset 1). Modules exhibiting a high topological overlap were visualized on a dendrogram using Dynamic Tree Cut algorithm (Fig. 1a).

### Module preservation and validation reveals co-regulated, preserved common genes between different cancers

Module preservation analysis was carried out at two levels to probe the robustness of conservation or stability–.

#### Identification of common enriched modules and gene sets across different cancer types

To address our hypothesis that some modules and genes are conserved across eleven different cancers and possibly define common functions, we evaluated the co-expression relationships between modules by applying module preservation statistics *Z summary* and *medianRank* across the eleven cancers (Fig. 1b; Supplementary Dataset 2; Supplementary Table S1). Four cancers *viz.* BRCA, COAD, OVCA and READ expressed strong module preservation, with OVCA exhibiting maximum co-expression with BRCA-COAD-GBM-LUAD-READ, followed by BRCA with COAD-LUAD-OVCA-READ-GBM; COAD with BRCA-OVCA-READ and READ with BRCA-COAD-OVCA (Fig. 1b). Moderate module preservation was expressed by two cancers *viz.* GBM (with OVCA-LUAD-BRCA-COAD) and LUAD (with BRCA-COAD-GBM-OVCA). However, KIRC, KIRP, LGG, LUSC and UCEC modules show very poor preservation with any cancer. Further extracting details of preserved modules and genes from the contingency table (Supplementary Dataset 2; Supplementary Table S2) revealed three strongly conserved modules in BRCA-COAD-OVCA-READ, and one of which not only preserved but was in fact, the only module conserved in GBM-LUAD (Fig. 1c). This affirms the BRCA-OVCA-COAD-READ group as expressing strong module preservation, while GBM-LUAD present moderate module preservation. One common gene sets was associated with each of the 3 preserved modules *viz.* Set 1 (n = 161), Set 2 (n = 50) and Set 3 (n = 44) in the BRCA-OVCA-COAD-READ group, while a subset of Set 1 genes (n = 55; referred to as the Set 1-s) was conserved in the GBM-LUAD (Fig. 1d; Supplementary Dataset 2; Supplementary Tables S2, S3). Despite a high preservation with OVCA-LUAD-BRCA-COAD, GBM stratifies into the moderate group due to conservation of a single module. Together, these results provided a first definitive proof of conserved modules and genes across different cancers.

#### Identification of robustly conserved modules across different validation datasets

Expression datasets other than the TCGA available in the public domain for the six conserved cancers were used to validate robustness of network module identification across different platforms and various cohorts (GEO^24^; Supplementary Dataset 3; Supplementary Table S1). Although this analysis revealed variations in numbers of WGCNA modules and genes, high preservation was evident across different datasets representing the same cancer type (Supplementary Dataset 3; Supplementary Figs S1-S2; Supplementary Dataset 3; Supplementary Table S2). Set 1 (n = 161) and Set 2 (n = 50) genes were moderately to strongly conserved in all validation datasets within the BRCA-COAD-OVCA-READ group, while Set 1-s genes were strongly conserved in the GBM-LUAD group (Supplementary Dataset 3; Supplementary Fig. S3); poor validation of Set 3 genes led to their exclusion from further analyses.

### Important hub genes and transcriptional regulators are identified within each preserved gene set

**Figure 2:**
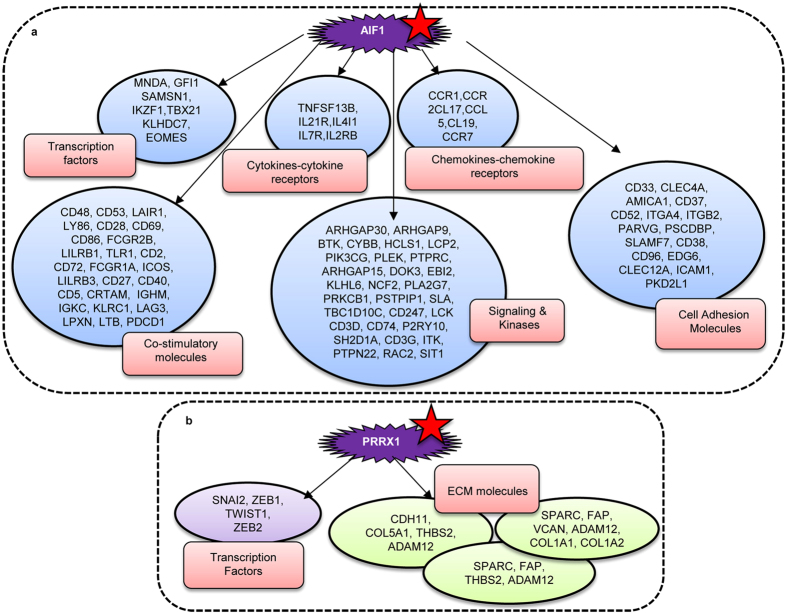
AIF1 and PRRX1 as master regulators interact with several genes to mediate specific functionalities.

Literature annotation identified Set 1 and Set 1-s to comprise of Transcription Factors (TFs), co-stimulatory molecules, cytokines-cytokine receptors, chemokines-chemokine receptors, cell adhesion molecules and signaling kinases involved in inflammatory and immune responses in the context of cancer development and progression, while Set 2 genes include TFs and ECM molecules involved in cell adhesion, angiogenesis, migration and invasion (Fig. 2). We also probed co-expression networks that could indicate interactions and similar functionalities between enriched genes within WGCNA. Identifying significant genes within each set as those hubs with at least ten interacting partners revealed several specific important hubs within Set 1 and Set 2 genes for all 6 cancers (Supplementary Dataset 4; Supplementary Fig. S1; Supplementary Table S1). Interestingly, although direct interactions between Set 1 and Set 2 hubs did not exist in any cancer, cross-talk through specific interactors was identified in OVCA (Supplementary Dataset 4; Supplementary Fig. S1). We scanned the interacting networks to find key TFs that could be crucial in regulating gene expression during development, differentiation and function of immune cells and/or tumor cell metastases identified AIF1, IKZF1, MNDA, SAMSN1, EOMES, GFI1 and KLHDC7B as significant TF-hubs within Set 1/Set 1-s gene networks in the six conserved tumor types (Supplementary Dataset 4; Supplementary Table S2). These TFs are known to mediate specific biological functions in immune regulation and/or cell migration. TBX21 (Tbet), EOMES and IKAROS regulate the differentiation and function of inflammatory Th1 cells; GFI modulates the differentiation of thymic regulatory CD4 T cells, while MNDA controls TRAIL induced apoptosis of granulocyte-macrophage progenitor cells. Th1 cells are known to inhibit cancer growth whereas Tregs makes the tumor microenvironment more immunologically suppressive. SAMSN1 is reported as being highly expressed in GBM tissue and has been suggested as a prognostic marker^25^. The Evi group of transcription factors are known to inhibit granulocyte—erythroid lineages and promote megakaryocytic differentiation^26^. Although KLHDC7B is reported to be highly expressed in breast cancer, its functions are not well studied^27^. Enriched Set 2 TFs (PRRX1-SNAI1-SNAI2-ZEB1-ZEB2-TWIST1) are extensively studied in the context of cell migration during embryonic development and tumor metastases and mediate EMT^28^,^29^,^30^,^31^,^32^. These TFs are also additionally known to be associated with other features besides EMT including oncogenic transformation, resistance to apoptosis and senescence, cancer cell stemness, and can also promote tumor angiogenesis^33^,^34^,^35^,^36^.

Amongst the Set 1 TFs, AIF1 appears to be the most significant hub with maximum network interactions and strong correlation with its interacting partners in the BRCA-COAD-OVCA-READ-GBM-LUAD group (Supplementary Dataset 4; Supplementary Fig. S2). Thus AIF1 within its hub may control expression of other TFs responsible for the development and function of immune cells involved in tumor growth. It is known to regulate several important co-stimulatory molecules in the tumor microenvironment that plays vital role in biological processes like immune response, inflammation, cell survival, apoptosis, proliferation and angiogenesis, is involved in regulating expression of cytokine and cytokine receptors that play a crucial role innate as well as adaptive inflammatory host defenses, apoptosis, angiogenesis, cell growth *etc.* AIF1 also controls key chemokine receptors involved in migration of immune cells into the tumor micrenvironment, and can also modulate the various kinases and signaling molecules; it is involved in inflammatory responses, promotes cell proliferation via activation of NFκB/cyclin D1 pathway and facilitates tumor growth which implicate its association with immune modulation^37^,^38^. In addition to immunomodulation, our data indicates that several important cell adhesion molecules such as *ICAM1, CD38, CD33, CD37* and *CD52* may be regulated by AIF1. Further, such regulation need not be restricted only to cancer cells but could extend further to infiltrating immune and stromal cells towards making the tumor microenvironment favorable for growth and metastases. Such direct or indirect interactions with several key regulatory genes towards regulation of differentiation and function of immune cells, controlling inflammation and promoting a tolerogenic microenvironment during tumor establishment makes AIF1 a candidate ‘master’ gene regulator of immunomodulation.

Within the Set 2 interacting networks, PRRX1 was the lone significant, large conserved TF-hub in the BRCA-COAD-OVCA-READ strongly preserved tumors although other TFs including EVI2A, EVI2B, TBX21, SNAI1, SNAI2, ZEB1, ZEB2 and TWIST1 were also identified from their extended interactions with the primary important hubs. Like AIF1, PRRX1 expression strong correlated with its interacting partners, several of which were conserved across the four cancers (Supplementary Dataset 4; Supplementary Fig. S2). PRRX1 expression is implicated in the induction of EMT, while lowered expression confers colonization and stemness abilities^39^. Our results corroborate these findings by revealing its interactions with several EMT-TFs (SNAI1, SNAI2, ZEB1, ZEB2, TWIST1), ECM components (ADAM12, CDH11, COL5A1, THBS2, SPARC, FAP, VCAN, COL1A1, COL1A2) and also with ZNF469, which is a TF associated with collagen synthesis and organization^40^. A possible regulatory role for PRRX1 is thus assigned in the regulation of metastases. Effectively, a cumulative effect of inflammatory and tolerogenic immune responses in the tumor microenvironment controlled by AIF1 and PRRX1 are identified to dictate tumor growth and metastasis. These findings together very importantly, suggests that the two conserved gene sets drive biological functions contributing to some of the hallmarks of cancer^41^. To confirm that regulation of immunomodulation and metastasis by master regulators AIF1 and PRRX1 were not specific to the TCGA datasets, we independently validated gene interactions regulated by Set 1/Set 1-s and Set 2 for six cancers in in other datasets of six cancers (Supplementary Dataset 3; Supplementary Table 1). Consistent with the TCGA-based results, gene interactions in these validation datasets affirmed regulation of genes and TFs involved in immune responses and metastasis to be governed by AIF1 and PRRX1 respectively (Supplementary Dataset 5).

### Stratification with Set1/Set 1-s and Set 2 genes correlates with clinical outcome

We further defined the 44 common significant hub markers amongst the preserved gene sets (36 and 8 genes from Set 1 and Set 2 respectively) across the 4 strongly preserved cancers (BRCA-COAD-OVCA-READ) as classifiers and applied these in the stratification of TCGA tumor sample datasets into 4 classes (Supplementary Dataset 4; Supplementary Table S3). In each cancer, Class 1 represents expressed classifiers of both gene sets; Class 2 and Class 3 are associated with upregulated Set 1 and Set 2 classifiers respectively while Class 4 represents downregulated classifiers of both sets. The 2 moderately preserved cancers (GBM-LUAD) were stratified into 2 classes using 33 Set 1-s gene classifiers. GSEA affirmed upregulation of Set 1 associated pathways in Class 1—Class 2 samples and their downregulation in Class 3—Class 4 for BRCA-COAD-OVCA-READ. In GBM-LUAD, Class 1 and Class 2 samples exhibited upregulated and downregulated pathways associated with Set 1-s genes (Supplementary Dataset 6; Supplementary Figs S1-S2; Supplementary Dataset 6; Supplementary Tables S1-S2). Set 1 & Set 1-s genes influenced pathways of inflammatory responses and are related with cancer development and progression. Similarly, Class 1 and Class 3 across all BRCA-COAD-OVCA-READ were positively associated with Set 2 driven pathways whereas class 2 and 4 show a negative association (Supplementary Dataset 6; Supplementary Fig. S3; Supplementary Dataset 6; Supplementary Table S3). Three Set 2 pathways including ECM-receptor interactions (govern tissue and organ morphogenesis to maintain cell and tissue structure and function), focal adhesion (cell motility, proliferation, differentiation, regulation of gene expression and cell survival) and integrin 1 (adhesion receptors in cell-extracellular matrix interactions) were common to all 4 cancers.

**Figure 3:**
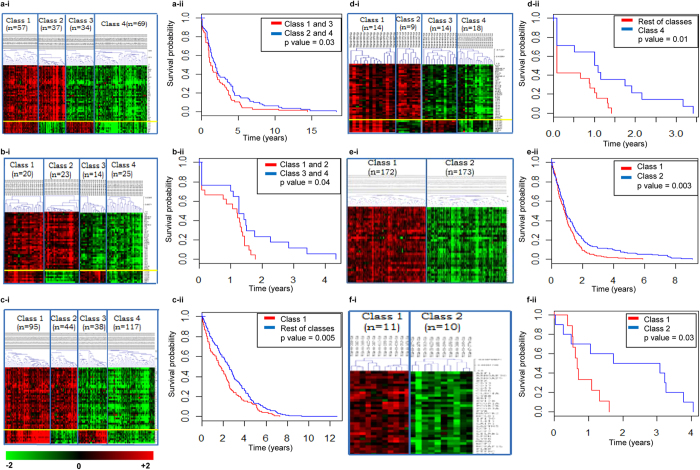
Heat map representation of 44 classifiers (Set 1: n = 36; Set 2: n = 8) expression based stratification into four classes and associated Kaplan Meier analysis for survival in (**a**) BRCA (**b**) COAD (**c**) OVCA (**d**) READ; and similarly for 33 Set 1-s classifier expression based stratification into two classes in (**e**) GBM and (**f**) LUAD.

To find a correlation between these classifiers and patient prognosis, expression of Set 2 classifiers appears to be significantly correlate with poor clinical outcome in BRCA patients (Classes 1 and 3 *vs.* Classes 2 and 4; Fig. 3a-i,a-ii) while Set 1 classifier expression in COAD correlate significantly with poor clinical outcome (Classes 1 and 2 *vs.* Classes 3 and 4; Fig. 3b-i,b-ii). In OVCA, Class 1 patients in which classifiers of both gene sets are upregulated, present the worst survival as compared to any other class (Fig. 3c-i,c-ii), that could possibly result from complementation of biological functions to drive aggressive disease and poor patient survival. On the other hand, Class 4 READ patients in which classifiers of both gene sets are downregulated show best prognosis as compared to any other class (Fig. 3d-i,d-ii), suggestive that expression of classifiers from either of the two gene sets can contribute to adverse prognosis. Expression of the 33 Set 1-s classifiers in GBM and LUAD Class 1 tumors associates them with poor prognosis as compared to Class 2 patients in which these genes are downregulated (Fig. 3e-i,e-ii,f-i,f-ii). Taken together, such stratification identifies differential correlation between expression of classifiers and patient survival.

This class-associated prognosis enticed us to further winnow out significant cancer-specific risk genes (prognostic factors) within the Set 1, Set 2 and Set 1-s genes (methods detailed in Supplementary-Dataset 7). Thus 23 risk genes were identified in BRCA patients and all of them were associated with Set 2 and included 4 classifiers *viz. COL5A2, FAP, COL5A1* and *ADAM12* (Supplementary Dataset 7; Supplementary Fig. S1; Supplementary Dataset 7; Supplementary Table S1; p ≤ 0.01). In case of COAD patients, 18 Set 1 genes were identified as risk genes, 5 of which *viz. PLEK, CLEC4A, LCP2, ITK* and *CD53* were classifiers (Supplementary Dataset 7; Supplementary Fig. S2; Supplementary-Dataset 7; Supplementary Table S1; p ≤ 0.01). 12 risk genes from Set 1 and Set 2 (n = 2 and n = 10 respectively) were predicted for OVCA cancer, none of which were amongst the classifiers (Supplementary Dataset 7; Supplementary Fig. S3; Supplementary-Dataset 7; Supplementary Table S1; p ≤ 0.01). 39 risk genes were predicted in READ patients (Supplementary Dataset 7; Supplementary Fig. S4; Supplementary Dataset 7; Supplementary Table S1; p ≤ 0.01) 30 of which were from Set 1 (including 15 classifiers *viz. PTPRC, CD33, CLEC4A, CYBB, AIF1, CD53, PLEK, EVI2A, CD74, CD48, LCP2, ITGB2, CD52, LAPTM5, ARHGAP15*) and 9 from Set 2 (single classifier *CDH11*). This further strengthens the possibility that complementation of biological functions between Set 1 and Set 2 genes may work against survival in OVCA and READ. 24 of the 37 predicted risk genes in GBM were classifiers (Supplementary Dataset 7; Supplementary Fig. S5; Supplementary Dataset 7; Supplementary Table S1; p ≤ 0.01), while 23 of the 26 predicted risk genes in LUAD were classifiers (Supplementary Dataset 7; Supplementary Fig. S6; Supplementary Dataset 7; Supplementary Table S1; p < 0.05).

### Common risk genes effectively predicts survival of cancer patients

**Figure 4:**
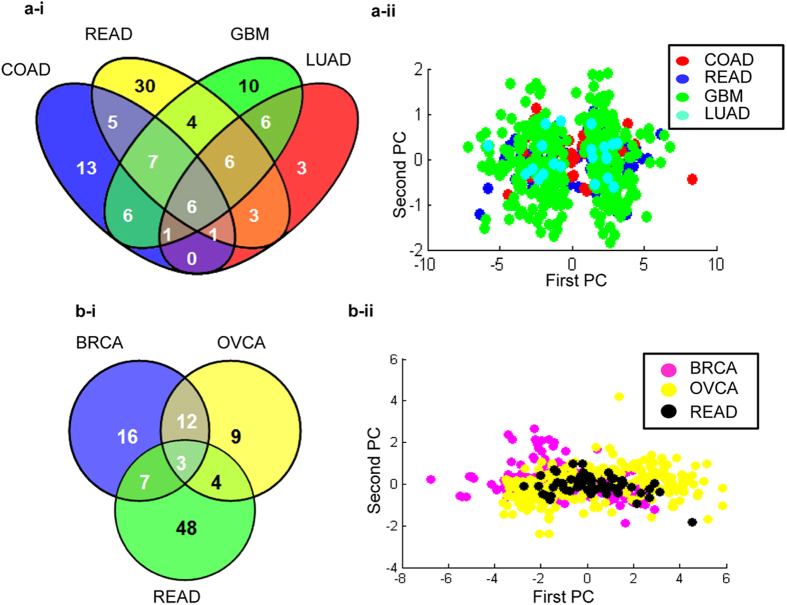
(**a**-**i**) Venn diagram representing common risk genes (p < 0.05); (**a-ii**) Projection of tumor samples onto the plane defined by the first and the second principal components using 6 risk genes for COAD-READ-GBM-LUAD group, High positive shift along first PC suggests overall decrease in gene expression whereas negative shift denotes overall increase in gene expression; (**b**-**i)** Venn diagram and (**b**-**ii**) PCA plot for samples of BRCA-OVCA-READ group.

The above risk signatures unique for each individual cancer involve a large number of genes for establishing prognosis. We hypothesized that a practical approach towards simplifying the same without compromising the predictive potential may be possible by applying selective common genes as opposed to an extensive panel of cancer-specific genes that would be a convenience in moving prognostic predictions to a next level of applications. To evaluate this, we further identified six Set 1/Set 1-s common risk factors in COAD, OVCA, READ, GBM and LUAD (*PLEK, LCP2, CD53, MNDA, NCF2, CYBB*; p < 0.05; Fig. 4a-i; OVCA was an outlier and failed to demonstrate any commonality in this set of genes). Similarly three Set 2 genes were identified as being common to BRCA-OVCA-READ (*WISP1, CTSK, ADAM12*; p < 0.05; Fig. 4b-i). Principal component analysis (PCA) of these risk genes to observe their correlation and joint behavior across patients of COAD-READ-GBM-LUAD and BRCA-OVCA-READ cancers was carried out using first three principal components (PCs) that capture 96% of expression variance. Gene weights for first PC have same sign and similar values and represent a fairly uniform shift in the overall expression (Supplementary Dataset 7; Supplementary Table S2). Further projection of COAD-READ-GBM-LUAD or BRCA-OVCA-READ tumor samples onto a plane defined by the first two PCs stratified these into two discrete clusters based on a either a positive and negative shift along the first PC that represents differential survival (Fig. 4a-ii,b-ii). The first three PCs account for 99% of expression variance for genes, and as above, similar sign and weights for first PC reflect a uniform shift in overall expression (Supplementary Dataset 7; Supplementary Table S2).

**Figure 5:**
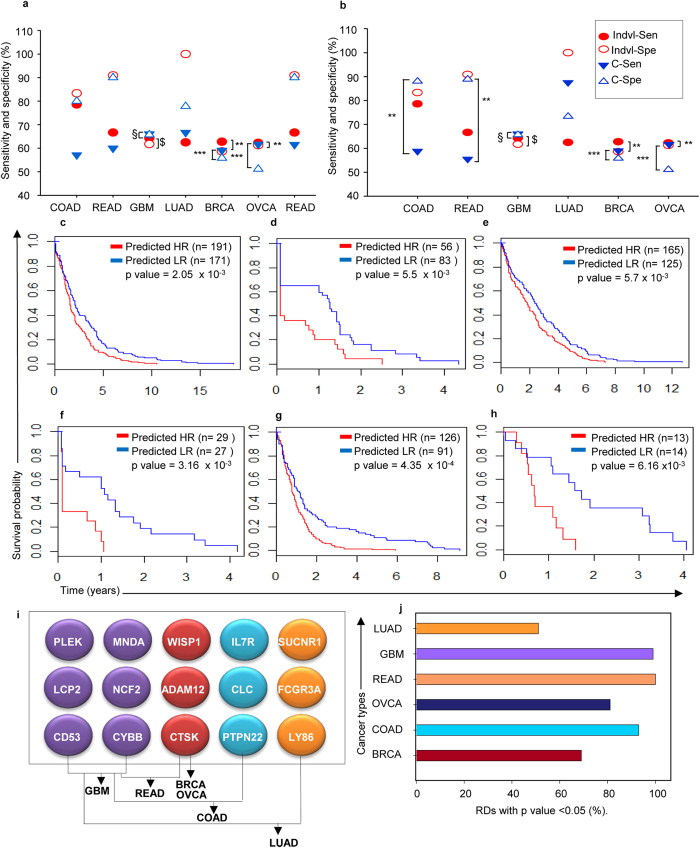
(**a**,**b**) Comparison of prognostic efficacies (Sen-Sensitivity, Spe-Specificity) between (**a**) individual (indvl) vs. common (**c**) risk signatures and (**b**) individual vs. 15-gene signature for six cancers, $-p < 1 × 10^−8^, §-p < 1 × 10^−7^; (**c**–**h**) K-M plots of survival in predicted risk groups based on differential expression of specific gene subsets within the 15-gene signature in BRCA, COAD, OVCA, READ, GBM or LUAD cancers in HR (high risk) and LR (low risk) samples; (**i**) GBOCRL-IIPr chip for the 15-gene signature (first 6 genes panel (left to right)- 6 common Set 1 risk genes, second 3 genes panel—3 common Set 2 risk genes, third 3 genes panel—3 most significant genes from COAD, last 3 genes panel—3 most significant genes from LUAD); (**j**) Graphical representation of risk assessment of GBOCRL- IIPr panel through re-sampling in 100 random datasets of the six cancers.

To further evaluate the efficacy of such practicality, comparative risk predictions between individual and common risk gene signatures in each cancer were determined. Thus, risk associations with gene expression were first established to (prediction of high or low risk; p < 0.05) followed by correlations with actual existing patient risk, that led to computation of sensitivity and specificity scores. In all cancers individual risk gene sensitivities and specificities ranged from 62–78% and 58–100% respectively, while the 6 common risk gene signature in exhibited 57–66% sensitivity and 65–80% specificity COAD-READ-GBM-LUAD and the 3 common risk gene signature ranges for sensitivity and specificity were 59–61% and 51–90% respectively in BRCA-OVCA-READ (Fig. 5a). Statistical evaluation of prognostic efficacies between individual and the common risk signatures using Mcnemar’s test assigned significance to prediction of high and low risk GBM-BRCA-OVCA over COAD-READ-LUAD patients (p < 0.05; Supplementary Dataset 7; Supplementary Tables S3-S4). Within the former group, the common risk signature prognostication was higher than that of individual risk genes for GBM patients, remained similar for in BRCA, and showed marginally lowered specificity in OVCA (Fig. 5a).

To further improve the prognostic prediction value for COAD-READ-LUAD, different approaches were tested. Since READ cancer patients showed similar prognostic efficacies for Set 1 and Set 2 genes, evaluating all 9 common genes (6 from Set 1 and 3 from Set 2) enhanced sensitivity and specificity to 55% and 88% respectively (Fig. 5b). On the other hand, in COAD and LUAD a combination of the 6 common risk genes from Set 1 with 3 most significant individual risk genes for each cancer type was tested, and observed to enhance prognostic efficacy (Fig. 5b; Supplementary Dataset 7; Supplementary Table S5). Kaplan-Meier analysis further supported these derivations since the defined gene subsets could successfully predict patients as being at either at a high or low risk and were further supported by Kaplan-Meier analysis (Fig. 5c–h; Supplementary Dataset 7; Supplementary Fig. S7). Thereby, 6 common Set 1 genes in GBM, 3 common Set 2 genes in BRCA-OVCA, 6 common Set 1 + 3 common Set 2 genes in READ, 6 common Set 1 + 3 most significant individual genes in COAD and LUAD are highly prognosticative. In conclusion, this 15-gene signature as identified through a comparative risk prediction between the individual and common risk genes represents in GBM-BRCA-OVCA-COAD-READ-LUAD (GBOCRL) cancers, the biological functions of immunomodulation and invasion (II) with a high prognostic (Pr) efficacy. This we termed as a GBOCRL-IIPr panel (Fig. 5i). As a final validation, we applied a random re-sampling strategy as described earlier^42^ within the TCGA data to generate 100 random datasets for each cancer type in which the GBOCRL-IIPr panel was further evaluated (Supplementary Dataset 8; Supplementary Table 1). The robust predictive power achieved in this reassessment confirmed the statistical significance of GBOCRL-IIPr genes in these six cancers (Fig. 5j).

## Discussion

Prognostic biomarkers are realized to be one of the central features in the concept of “personalized cancer therapy”, due to their informative association with clinical outcomes. However, translational from a preliminary identification to application in the clinic is fraught with several obstacles. The utility of screening a set of genes for their expression to predict patient prognosis indeed, deems such an approach to be commercially viable only if the incidence of that particular cancer is significantly high. Thus, the derivation of a common prognostic panel of biomarkers could provide a promising advantage in addressing such practicalities in the field of biomarkers.

Studies relating to prognostic markers have largely relied on individual gene identification, that are often divorced from correlation with biological tumor behavior^43^. Within the diversity of systems analysis, gene co-expression studies such as WGCNA offer a robust, unbiased means of establishing cancer-specific networks that can be used to define and stratify tumors based on biological functions. In the present study, we first hypothesized the preservation of common biological networks across different cancers that might further find concordance with shared tumor cell properties such as those defined as hallmarks of cancer^41^. After affirming the presence of conserved genes and modules across six cancers, we applied their differential expression profiles to achieve patient stratification and establish class-associations with survival. This led us to derive a set of cancer-specific risk genes that were associated with patient prognosis. From an observation that some of these genes were strikingly preserved across the cancer types under study and through a rigorous evaluation of the common vs. individual risk genes, we formulated a panel of 15 prognostic markers termed as the GBOCRL (cancer types)—IIPr panel (biological functions). Since cancer samples are full of random gene expression signals, we validated this panel for its predictive power and achieved a significant confirmation over 100 random datasets generated by re-sampling as described before^42^, to provide a strong validation to the GBOCRL—IIPr panel.

Conserved biological pathways between breast and ovarian cancer are known^44^, while some of the identified prognostic markers are also previously reported as cancer prognostic factors. *CTSK* expression is reported to govern breast tumor progression and prognosis by promoting extracellular matrix degradation and angiogenesis^45^; *CD53* expression was significantly associated with distant metastasis-free survival in ER^−^ breast cancer patients^46^; *ADAM12* is proposed as a biomarker and drug target in breast cancer^47^. Significantly, although we identified two master regulators *viz.* AIF1 and PRRX1 within conserved biological networks, and which are possibly associated with the hallmarks of tumor evasion/immunomodulation and metastases respectively, neither these nor any of the other important hub genes were reflected as prognostic biomarkers. However it is suggest that since the 15 gene signature is regulated by AIF1 and PRRX1, these two TFs may be considered as novel therapeutic targets.

In conclusion, screening for expression of the GBOCRL- IIPr panel may further be commercialized as a microarray platform formulation and extend our findings to the clinical prognosis. Such prognosis based stratification of cancer patients at the time of diagnosis can be very helpful in formulating treatment strategies.

## Materials and Methods

A summary of methodology applied for derivation of the GBOCRL-IIPr prognotic panel is outlined in Supplementary Fig. 1.

### Microarray expression datasets for different cancers

Cancer types selected for this study are based on impact of the disease on overall public health and prognosis. Among men, cancers of the lung and bronchus, colorectum, kidney and renal account for ~ 39% of all estimated cancer deaths (lung and bronchus ~ 28%, Colon and rectum ~ 8%, kidney and renal ~ 3%), while in women, cancers of lung and bronchus, breast, colorectum, ovary, uterine corpus and brain account for ~ 60% of all estimated cancer deaths (lung and bronchus ~ 26%, breast ~ 15%, colorectum ~ 9%, ovary ~ 5%, uterine corpus ~ 3%, brain ~ 2%)^48^. These cancers are well represented in the TCGA datasets and hence we selected Level 3 normalized microarray gene expression data (UNC__AgilentG4502A_07) from TCGA database (https://tcga-data.nci.nih.gov/tcga/; 15/12/2012) of 11 cancer types viz. Breast invasive carcinoma (BRCA, n = 599), Colon Adenocarcinoma (COAD,n = 74), Glioblastoma multiforme (GBM,n = 604), Kidney renal Clear cell Carcinoma (KIRC, n = 72), Kidney renal papillary cell carcinoma (KIRP,n = 16), Brain lower grade Glioma (LGG, n = 27), Lung Adenocarcinoma (LUAD,n = 32), Lung squamous cell carcinoma (LUSC, n = 155), Ovarian Cystadenocarcinoma (OVCA, n = 598), Rectum adenocarcinoma (READ, n = 72), Uterine Corpus Endometrioid carcinoma (UCEC, n = 54). The TCGA data source however, because of the stringency of its selection may potentially harbour a bias effectively built into the system from inclusion of only those specimens with really high quality RNA qualified through the QC inclusion criteria, which may not be representative of all samples. However, computational analysis requires such stringency and hence the bias has been overlooked in the present analysis. Validation datasets for six cancer types (platform: Affymetrix) were downloaded from Gene Expression Omnibus database (GEO; http://www.ncbi.nlm.nih.gov/geo; 7/02/2013; Supplementary Dataset 3).

### Weighted gene co-expression network analysis (WGCNA) based module-construction, module preservation statistics and identification of conserved genes

WGCNA and module preservation statistics were carried out as described earlier^21^,^22^. Gene expression data clustered into different number of modules for each cancer type in WGCNA. Preservation across cancers was determined by applying module preservation statistics *Z summary* and *medianRank* across an 11 × 11 grid. Such analysis generates 121 graphical representations of these module preservation statistics (not displayed due to lack of space but can be provided on request). Percentage preservation was computed from the number of modules preserved across 11 cancer types [percentage of module preservation= (preserved modules/total number of modules * 100)] that is represented as a module preservation matrix (Fig. 1b). Strong preservation was derived for modules with Zsummary > 10-*medianRank* statistics < 10, whereas moderate preservation was defined as 2 < *Zsummary* < 10-*medianRank* statistics< 10. Module preservation differed across cancer types within the matrix making it an asymmetric one; this is better understood from the following example. 5 modules were conserved between BRCA and COAD (10 and 11 WGCNA modules respectively); thus BRCA vs. COAD module preservation = 5/10*100 = 50%, while that for COAD vs. BRCA = 5/11*100 = 45.45%. Thereby, module preservation differs across the matrix and assigns it an asymmetry. Significant genes conserved across modules were identified using cross tabulation statistics and Fisher-exact test.

### Identification of gene interactions among preserved sets

WGCNA was used to generate network modules and visualized using Cytoscape v2.8.3. Edge weighted force directed biolayout was used for generation of interaction networks for Set 1 and Set 2 genes of the BRCA-COAD-OVCA-READ group and Set 1-s genes of GBM-LUAD group wherein hub markers were defined as those genes with more than 10 interactions.

### Tumor sample classification and survival analysis

Stratification of BRCA-COAD-OVCA-READ tumor samples was carried out based on differential expression profiles of Set 1 and Set 2 genes, while Set 1-s genes were used to identify classes within GBM-LUAD. Further Kaplan-Meier (K-M) analysis was performed using R-based packages established association of each tumor class with survival; significance was determined by log rank tests.

### Assignment of biological significance using Gene Set Enrichment Analysis (GSEA)

GSEA^49^ was performed to identify biologic processes and signaling pathways regulated by Set 1-Set 2 genes for BRCA-COAD-OVCA-READ classes and Set 1-s genes for GBM-LUAD. Pathway networks were visualized using Enrichment map in Cytoscape_v2.8.3 (p < 0.05).

### Risk genes identification and computation of sensitivity and specificity

The identification of risk genes is detailed in Supplementary Dataset 7. Further, PERL code was used for assignment of high or low risk to patients using differential expression of significant risk genes (p < 0.05). Actual high and low risk patients were identified from TCGA data and thresholds defined as (≥Mean (survival period) for each cancer) –.

- high risk <3 years> low risk for BRCA and OVCA.
- high risk <1 year> low risk for COAD, READ and LUAD.
- high risk <2 years> low risk for GBM.

True Positive (TP), False Positive (FP), True Negative (TN) and False Negative (FN) events were identified by comparing both approaches. Sensitivity (TP/TP + FN) and specificity (TN / TN + FP) of risk genes were computed for each cancer types and Mcnemar’s test was used to identify significant prognostic efficacies^50^.

### Random resampling and revalidation of panel genes

Resampling in the six cancers (from TCGA dataset—Supplementary Dataset 1; Supplementary Table 1) was carried out as described earlier^42^. Briefly, 100 random datasets (RDs) each for BRCA, OVCA and GBM (200 samples), COAD (120 samples), READ (50 samples) and LUAD (20 samples) were generated using R software. Differential expression pattern of risk genes (GBOCRL- IIPr panel; Fig. 5i) were used for assignment of low and high risk of the patients. K-M analysis performed with log rank test indicated that differential expression pattern of risk genes significantly correlated with prediction of low and high risk groups within the RDs (p value < 0.05; Supplementary Dataset 8; Supplementary Table 1).

## Additional Information

**How to cite this article**: Khirade, M. F. *et al.* Derivation of a fifteen gene prognostic panel for six cancers. *Sci. Rep.* **5**, 13248; doi: 10.1038/srep13248 (2015).

## Supplementary Material

Supplementary Information
