# Supplementary material for: Derivation of a fifteen gene prognostic panel for six cancers
Source: Sci Rep. 2015 Aug 14;5:13248. doi: 10.1038/srep13248 (PMC4536526; doi:10.1038/srep13248)
Supplement: Supplementary Information [file srep13248-s1.pdf]

Supplementary Information

Derivation of a fifteen gene prognostic panel for six cancers

Mamata F.Khirade[1], Girdhari Lal, Sharmila A. Bapat[1,2]

[1]National Centre for Cell Science, NCCS Complex, Pune 411007, INDIA

[2]Corresponding author: Sharmila A. Bapat, National Centre for Cell Science, NCCS Complex, Pune 411007, INDIA, Tel.91-020-25708074, email:sabapat@nccs.res.in

Running Title: Derivation of GBOCRL-IIPr panel

Supplementary Figure 1. Flow-chart of analytical approach

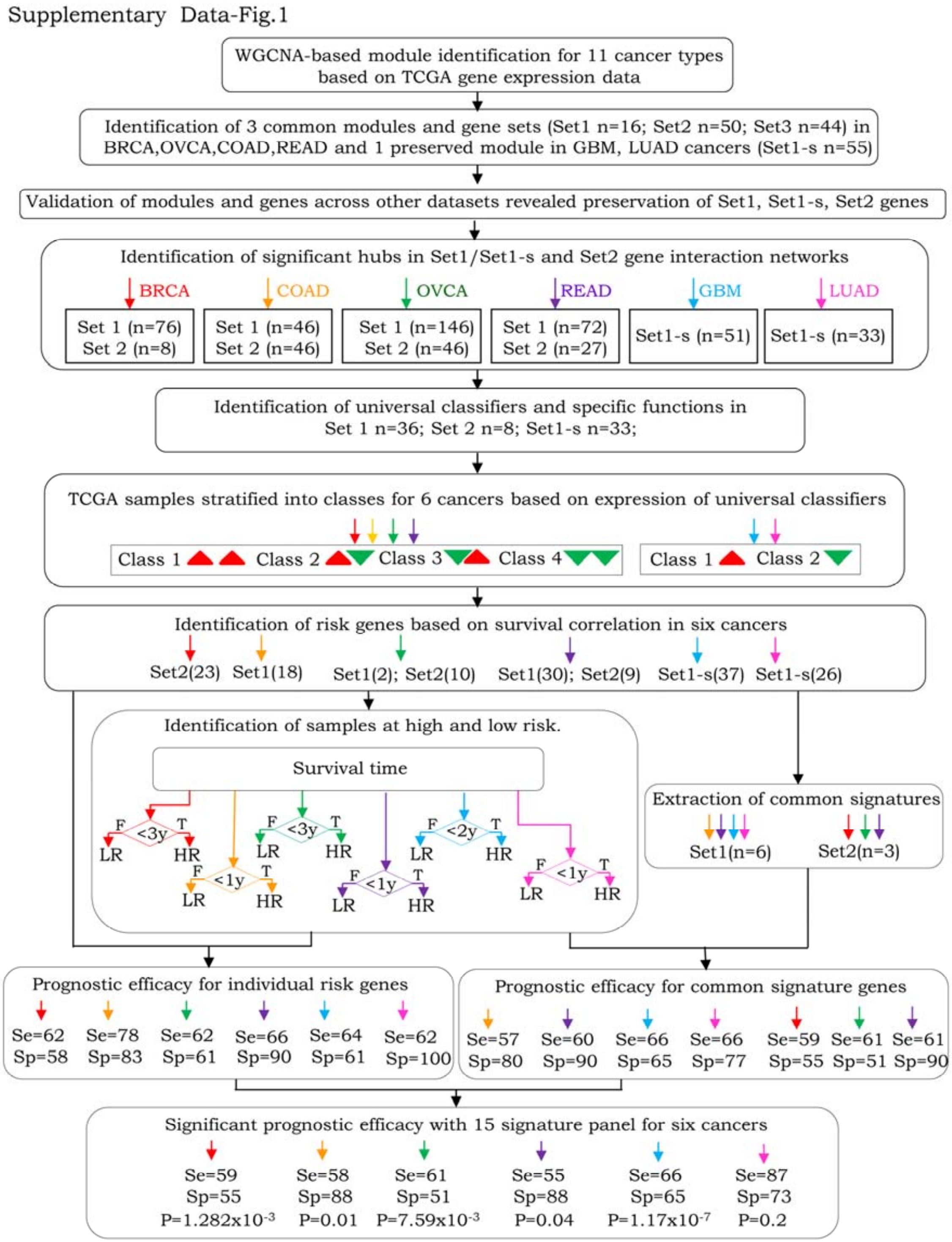

Supplementary Dataset1. Microarray expression dataset analysis of different cancers for Weighted Gene Co-expression Network Analysis (WGCNA)

Microarray gene expression data (UNC__AgilentG4502A_07) was downloaded from The Cancer Genome Database (TCGA; https://tcga-data.nci.nih.gov/tcga/), for 11 cancer types viz. Breast invasive carcinoma (BRCA), Colon Adenocarcinoma (COAD), Glioblastoma multiforme (GBM), Kidney Renal Clear cell Carcinoma (KIRC), Kidney renal papillary cell carcinoma (KIRP), Brain Lower Grade Glioma (LGG), Lung Adenocarcinoma (LUAD), Lung squamous cell carcinoma (LUSC), Ovarian Cystadenocarcinoma (OVCA), Rectum adenocarcinoma (READ), Uterine Corpus Endometrioid carcinoma (UCEC). Median absolute deviation (MAD) analysis was carried out using the Biometric Research Branch tool (BRB;1) to identify statistically significant differentially expressed genes in a tumor-specific manner of a total of 17,814 genes present on the platform. Effectively, this reduced the number of genes being considered in the analysis to a range of 4343 – 7206 genes in the different cancer types (each gene being expressed at a minimum 1.5 fold difference of $\log_2$ intensity values as compared with its median value in at least 20% of samples within a specific cancer type; $p<0.05$; Supplementary Dataset1-Table1).

Supplementary Dataset1-Table1. Median Absolute Deviation analysis and WGCNA module identification

Cancer Type	No. of samples	No. of differentially expressed genes following MAD analyses	β-values of correlation matrix in WGCNA	No. of modules identified in WGCNA
BRCA	599	7169	12	10
COAD	174	6541	14	11
GBM	604	7206	6	13
KIRC	72	6173	14	9
KIRP	16	4343	14	23
LGG	27	5356	9	13
LUAD	32	5250	9	18
LUSC	155	6578	18	5
OVCA	598	7046	3	12
READ	72	5886	12	6
UCEC	54	5842	20	9

We further subjected the differentially expressed gene lists obtained following MAD analysis to weighted gene co-expression Network construction (WGCNA; 2,3). In co-expression networks, nodes represent genes and connection strengths between nodes are determined by pairwise correlations between expression profiles. Connection strengths between two genes in weighted networks use soft thresholding of the Pearson correlation matrix that leads highly robust network construction. In our study, a pair-wise Pearson correlation matrix was computed for each set of differentially expressed genes. Following this, an adjacency matrix was calculated by raising the power of correlation (Beta- β value) that produces a weighted correlation matrix. β values were selected using scale free topology approach (r^2 curve level highest at β value). The adjacency matrix was useful for calculating a network of topological overlap (TO), followed by average linkage hierarchical clustering of genes using TO as input. Network modules (clusters of highly co-expressed genes) were identified for each cancer types (Supplementary Dataset1-Table1).

References

1. Simon R, Lam A, Li MC, Ngan M, Menenzes S, Zhao, Y. Analysis of gene expression data using BRB-ArrayTools. Cancer Inform 2007;3:11-17.
2. Langfelder P, Horvath S. WGCNA: an R package for weighted correlation network analysis. BMC.Bioinformatics 2008;9:559.
3. Ghazalpour A, Doss S, Zhang B, Wang S, Plaisier C, Castellanos R, et al. Integrating genetic and network analysis to characterize genes related to mouse weight. PLoS.Genet 2006;2:e130.

Supplementary Dataset2. Module preservation statistics and Identification of conserved genes

Supplementary Dataset2-Table1. Summary matrix indicating module preservation through stability of gene co-expression across 11 cancer types

Cancer Type	BRCA	COAD	GBM	KIRC	KIRP	LGG	LUAD	LUSC	OVCA	READ	UCEC
**BRCA**	**10**	**5**	**3**	2	1	2	**4**	0	**4**	**4**	0
**COAD**	**5**	**11**	**3**	2	1	2	**3**	0	**4**	**6**	0
**GBM**	**3**	**3**	**13**	3	1	4	**4**	1	**7**	**2**	0
**KIRC**	2	2	3	**9**	1	1	2	0	2	2	0
**KIRP**	1	1	1	1	**23**	1	2	0	1	1	0
**LGG**	2	2	4	1	1	**13**	2	0	2	1	0
**LUAD**	**4**	**3**	**4**	2	2	2	**18**	0	**5**	**2**	0
**LUSC**	0	0	1	0	0	0	0	**5**	0	0	2
**OVCA**	**4**	**4**	**7**	2	1	2	**5**	0	**9**	**3**	0
**READ**	**4**	**6**	**2**	2	1	1	**2**	0	**3**	**12**	0
**UCEC**	0	0	0	0	0	0	0	2	0	0	**6**

Module comparison identifies specific sets of conserved genes across strongly and moderately preserved cancers respectively

Genes conserved across modules were identified using cross-tabulation based module preservation statistics (Supplementary Dataset2-Fig.1). This contingency table indicated the high agreement between strongly preserved cancers BRCA, COAD, OVCA and READ and identifies three sets of modules that are strongly correlative between these cancers, while one module is moderately preserved in GBM and LUAD (Supplementary Dataset2-Tables 2,3). Further, within each of the three strongly conserved modules a group of preserved genes were identified and termed as Set1 (n=161) and Set2 (n=50) genes. Similarly within the moderately conserved module, a subset of Set1 genes (referred to as Set1-s,n=55) was identified as being preserved.

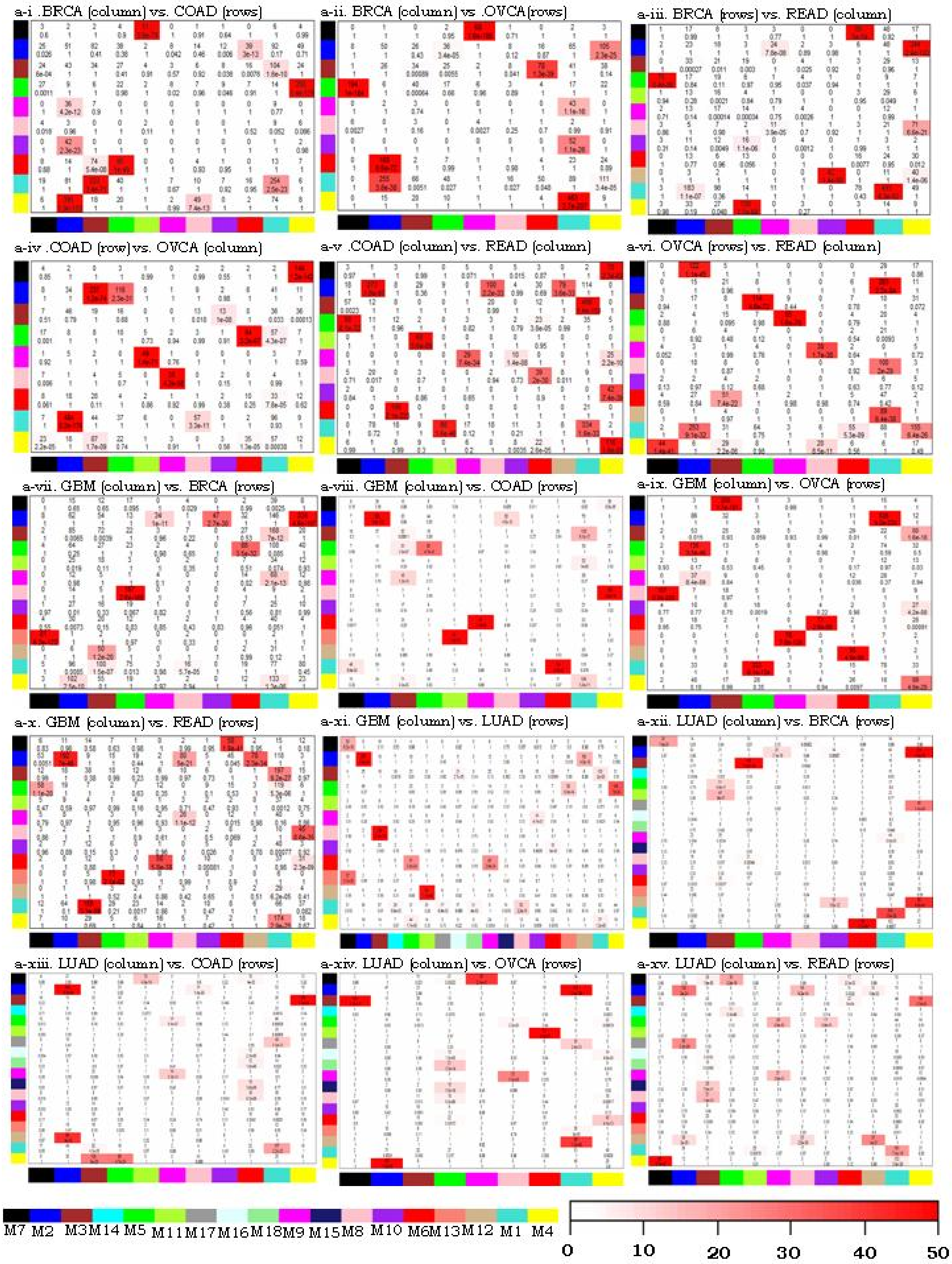

Supplementary Dataset2-Fig.1. Cross-tabulation based comparison of modules (defined as clusters) for 6 cancer networks represented as Contingency tables for comparison of - a-i,a-ii,a-iii. BRCA with COAD, OVCA and READ modules; a-iv,a-v. COAD with OVCA and READ modules; a-vi. OVCA modules with READ modules; a-vii,a-xi. GBM with BRCA, COAD, OVCA, READ and LUAD modules; a-xii,a-xv. LUAD with BRCA, COAD, OVCA and READ modules.

Supplementary Dataset2-Table2. Preservation of genes across corresponding modules between 6 cancer types (4 strongly and 2 moderately preserved cancers)

Strong preservation				Moderate preservation			
Comparison	Set1 genes	Set2 genes	Set3 genes	Comparison	Set1 genes	Set2 genes	Set3 genes
BRCA vs. COAD	395 (M4-M2)	95 (M6-M5)	51 (M7-M11)	GBM vs. BRCA	334 (M2-M4)	-	-
BRCA vs. OVCA	463 (M4-M1)	168 (M6-M2)	68 (M7-M9)	GBM vs. COAD	393 (M2-M2)	-	-
BRCA vs. READ	75 (M7-M5)	89 (M6-M7)	244 (M4-M2)	GBM vs. OVCA	536 (M2-M1)	-	-
COAD vs. OVCA	484 (M2-M1)	116 (M5-M2)	49 (M11-M9)	GBM vs. READ	192 (M2-M2)	-	-
COAD vs. READ	98 (M5-M7)	273 (M2-M2)	60 (M11-M5)	GBM vs. LUAD	270 (M2-M2)	-	-
OVCA vs. READ	122 (M2-M7)	65 (M9-M5)	263 (M1-M2)	LUAD vs. COAD	243 (M2-M2)	-	-
				LUAD vs. OVCA	302 (M2-M1)	-	-
				LUAD vs. BRCA	239 (M2-M4)	-	-
				LUAD vs. READ	-	-	-
**Signature genes**	**161**	**50**	**44**	-	**55**	-	-

Supplementary Dataset2-Table3. Identification of universal signature genes

Strong module preservation signature			Moderate module preservation signature		
**Set1 genes**	**Set2 genes**	**Set3 genes**	**Set1-s genes**	**Set2 genes**	**Set3 genes**
**161**	**50**	**44**	**55**	**-**	**-**
ACP5	ADAM12	AVP	AIF1		
ADAM8	BGN	BHLHB4	APOBEC3A		
AIF1	CDH11	CACNA1E	APOBEC3G		
AMICA1	COL10A1	CAMK2N2	ARHGAP30		
APOBEC3A	COL11A1	CASKIN1	ARHGAP9		
APOBEC3G	COL12A1	CDC42EP5	BTK		
APOBEC3H	COL1A1	CDH24	CD33		
ARHGAP15	COL1A2	COX6A2	CD37		
ARHGAP30	COL3A1	DLGAP3	CD53		
ARHGAP9	COL5A1	EN2	CD69		
ARHGDIB	COL5A2	FBXL17	CD86		
BTK	COL6A3	FCRLB	CLEC4A		
CCL17	COL8A1	FOXB1	CYBB		
CCL19	CORIN	GALR3	EBI2		
CCL5	CTHRC1	GP1BB	EVI2B		
CCR1	CTSK	GPR150	FCGR1A		
CCR2	DIO2	GPR153	FCGR2B		
CCR7	EDNRA	GPR156	FCGR3A		
CD2	F2R	GPR78	FYB		
CD247	FAP	GRIN2D	HAVCR2		
CD27	INHBA	HES7	HCLS1		
CD28	ITGA11	IFITM5	IFI30		
CD33	ITGBL1	KISS1R	IL4I1		
CD37	LOXL2	LCE1D	ITGB2		
CD38	LRRC15	LCE2D	KLHDC7B		
CD3D	MMP11	LCE5A	LAIR1		
CD3G	MXRA5	MAFA	LAPTM5		
CD40	NID2	MMP17	LCP1		
CD48	NOX4	MYOD1	LCP2		
CD5	P4HA3	NEUROG3	LILRB1		
CD52	POSTN	NLF2	LPXN		
CD53	PPAPDC1A	NPBWR1	LY86		
CD69	PPEF1	OR5L1	MNDA		
CD72	PRR16	OTUD7A	NCF2		
CD74	PRRX1	PCSK1N	NCKAP1L		
CD86	RCN3	PHOX2A	NPL		
CD96	SGIP1	POU3F3	P2RY6		

CHST11	SHOX2	psiTPTE22	PLEK		
CLC	SPARC	RHBDL1	PSCDBP		
CLEC12A	SPOCD1	SCRT2	PTAFR		
CLEC4A	SPON2	SOX18	PTPRC		
CORO1A	THBS2	TIP39	RAB42		
CRTAM	THY1	UTS2R	RGS18		
CST7	TMEM46	ZSCAN10	RNASE6		
CTSL1	TNFSF4		SAMSN1		
CYBB	VCAN		SIGLEC5		
DOCK8	WISP1		SIGLEC7		
DOK3	WNT2		SLA		
DPEP2	ZNF469		SLC37A2		
EBI2	CHSY-2		SUCNR1		
EDG6			TLR1		
EOMES			VMO1		
EVI2A			VNN2		
EVI2B			HLA-DQB1		
FCGR1A			HLA-DRB5		
FCGR2B					
FCGR3A					
FYB					
GFI1					
HAVCR2					
HCLS1					
HCST					
HPSE					
ICAM1					
ICOS					
IFI16					
IFI30					
IGHM					
IGKC					
IKZF1					
IL21R					
IL2RB					
IL4I1					
IL7R					
IRAK3					
ITGA4					
ITGB2					
ITK					
JAK2					
JAKMIP1					
KLHDC7B					
KLHL6					
KLRC1					
LAG3					
LAIR1					
LAMP3					
LAPTM5					
LCK					
LCP1					
LCP2					
LILRB1					
LILRB3					
LPXN					
LRRC8C					
LTB					
LY86					
MMP9					
MNDA					
NCF2					
NCF4					
NCKAP1L					
NPL					
P2RY10					
P2RY6					
PARVG					
PDCD1					
PIK3CG					

PKD2L1					
PLA2G7					
PLEK					
PRKCB1					
PSCD4					
PSCDBP					
PSTPIP1					
PTAFR					
PTPN22					
PTPRC					
PTPRCAP					
PVRIG					
RAB42					
RAC2					
RASSF4					
Rgr					
RGS1					
RGS18					
RNASE6					
SAMSN1					
SELPLG					
SH2D1A					
SIGLEC5					
SIGLEC7					
SIT1					
SLA					
SLAMF7					
SLC15A3					
SLC37A2					
SLFN11					
SP140					
SUCNR1					
TAGAP					
TBC1D10C					
TBX21					
TLR1					
TMEM149					
TNFSF13B					
TRAF3IP3					
TRAT1					
TRIM22					
TRPV2					
URP2					
VAV1					
VMO1					
VNN2					
WDFY4					
XCL2					
ZNF683					
HLA-DPB1					
HLA-DQB1					
HLA-DRB5					
IGKV1-5					
RP5-821D11.2					

Supplementary Dataset3. Validation of Module preservation across additional gene expression datasets of six cancer types

The following expression datasets available in the public domain (Gene Expression Omnibus-GEO; http://www.ncbi.nlm.nih.gov/geo) were used to identify robustness of network module across validation datasets (Supplementary Dataset3-Table1).

Supplementary Dataset3-Table1. Datasets used for validation of individual cancer network modules across different platforms

SR.NO	PLATFORM	DATASET RECORD	SAMPLE SIZE	REF.
	**BRCA**			
BRCA-VD1	Affymetrix Human Genome U133 Plus 2.0 Array	GDS4114	24	GSE26910
BRCA-VD2	Affymetrix Human Genome U133A Array	GDS4057	103	GSE22093
BRCA-VD3	Affymetrix Human Genome U133A Array	GDS4056	61	GSE23988
BRCA-VD4	Affymetrix Human Genome U133 Plus 2.0 Array	GDS3853	19	GSE21422
BRCA-VD5	Affymetrix Human Genome U133A Array	GDS3097	48	GSE5847
BRCA-VD6	Affymetrix Human Genome HG-U133A Array	GDS3096	47	GSE5847
BRCA-VD7	Affymetrix Human Genome U133 Plus 2.0 Array	GDS2635	30	GSE5764
	**COAD**			
COAD-VD1	Affymetrix Human Genome U133 Plus 2.0 Array	GDS4379	62	GSE35896
COAD-VD2	Affymetrix Human Genome U133 Plus 2.0 Array	GDS4296	174	GSE32474
COAD-VD3	ABI Human Genome Survey Microarray Version 2	GDS3756	42	GSE15781
COAD-VD4	Affymetrix Human Genome U95 Version 2 Array	GDS3384	23	GSE11237
	**OVCA**			
OVCA-VD1	Affymetrix Human Genome HG-U133A Array Plus 2.0 Array	GDS3592	24	GSE14407
OVCA-VD2	Affymetrix Human Genome HG_U95Av2 Version 2 Array	GDS2785	43	GSE7463
OVCA-VD3	Affymetrix Human Genome HG_U95Av2 Version 2 Array	GDS1381	18	GSE1926
	**READ**			
READ-VD1	ABI Human Genome Survey Microarray Version 2	GDS3756	42	GSE15781
READ-VD2	Affymetrix Human Genome HG-U133A Array Plus 2.0 Array	GDS4396	29	GSE28702
READ-VD3	Affymetrix Human Genome HG-U133A Array Plus 2.0 Array	GDS4393	54	GSE28702
	**GBM**			
GBM-VD1	Affymetrix Human Genome HG-U133A Array Plus 2.0 Array	GDS4467	35	GSE15824
GBM-VD2	Affymetrix Human Genome HG-U133A Array Plus 2.0 Array	GDS3885	92	GSE23806
GBM-VD3	Affymetrix Human Genome HG-U133A Array	GDS1975	85	GSE4412
GBM-VD4	Affymetrix Human Genome HG-U133A Array Plus 2.0 Array	GDS1962	180	GSE4290
	**LUAD**			
GBM-VD1	[Affymetrix Human Genome HG-U133A Array Plus 2.0 Array	GDS3257	58	GSE10072

Normalised data were subjected to Median Absolute Deviation (MAD) analysis to obtain significant differentially expressed genes (each expressing a minimum 1.5 fold difference with its median value in at least 20% of samples at log intensity variation $p<0.05$). WGCNA-based network modules were identified as described in Expanded View Dataset1. Such clustering of co-expressed genes revealed different numbers of modules in each cancer type (Supplementary Dataset3-Table2; Supplementary Dataset3-Fig.1).

Supplementary Dataset3-Table2. Network module identification in the validation datasets

Validation datasets	Output genes after MAD Analyses	β values of correlation matrix	Number of Modules	Strongly Validated modules	Moderately Validated modules
**BRCA-VD1**	6262	14	18	M4,M6	M1,M2,M3,M8,M9,M10
**BRCA-VD2**	6569	10	8	M1,M3,M4,M6	M2,M5,M9,M10
**BRCA-VD3**	6382	10	10	M2,M3,M4,M10	M1,M5,M6,M8,M9
**BRCA-VD4**	5599	20	12	M1,M4,M5	M2,M3,M6,M8,M9,M10
**BRCA-VD5**	4636	12	15	M1,M3,M4,M5, M6, M10	M2,M8,M9
**BRCA-VD6**	3706	10	11	M1,M3,M4,M5,M6, M10	M2,M8,M9
**BRCA-VD7**	7315	8	25	M1,M2,M6	M3,M4,M5,M9,M10
**COAD-VD1**	5470	6	11	M2,M3,M4,M5	M7,M8,M10
**COAD-VD 2**	5203	9	12	M3,M4	M2,M5,M6,M10
**COAD-VD 3**	4832	16	7	M3,M4,M5	M1,M2,M7,M8
**COAD-VD 4**	2876	14	10	M2,M3	M1,M4,M5,M6,M7,M8
**OVCA-VD1**	4414	20	6	-	M2,M3,M7
**OVCA-VD2**	3766	20	6	M1,M7	M2,M4,M5,M6
**OVCA-VD3**	3784	4	10	M7	M1,M2
**READ-VD1**	5010	10	7	M1,M7	M2,M4,M6,M10,M11
**READ-VD2**	7095	4	25	M2	M1,M4,M6,M7,M8,M9, M11, M12
**READ-VD3**	4329	9	9	M1,M7	M2,M6,M8,M11,12
**GBM-VD1**	4943	20	11	M2,M3,M4,M5, M8, M11	M10,M12,M13
**GBM-VD2**	1977	20	4	M4,M8	M1,M2,M5,M6,M9,M10
**GBM-VD3**	6685	14	8	M2,M3,M4,M5, M8, M11	M6,M10
**GBM-VD4**	100053	14	8	M2,M3,M5,M8, M11	M4,M7,M10
**LUAD-VD1**	4752	8	8	M2,M3,M4,M12	M1,M13,M14,M17

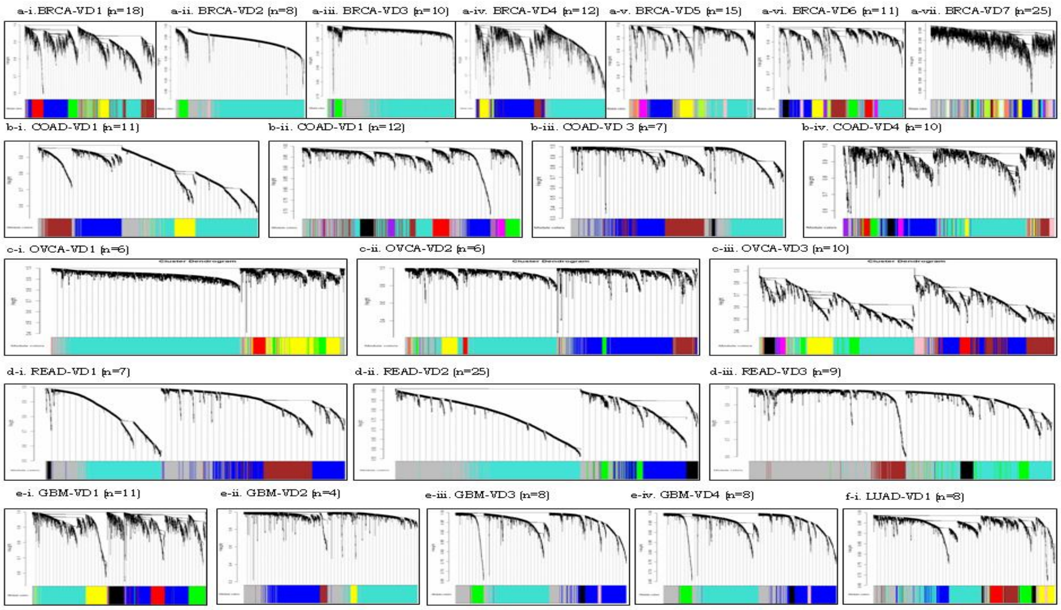

Supplementary Dataset3-Fig.1. Cluster dendrograms validation datasets, a-i-a-vii. BRCA-VD1 to BRCA-VD7, b-i-b-iv. COAD-VD1 to COAD-VD4, c-i-c-iii. OVCA-VD1 to OVCA-VD3,d-i-d-iii. 3 READ-VD1 to READ-VD3, e-i-e-iv. GBM-VD1 to GBM-VD3, f-i. LUAD-VD1; each dendrogram represents clustering of co-expressed genes in which modules are represented in distinct colors.

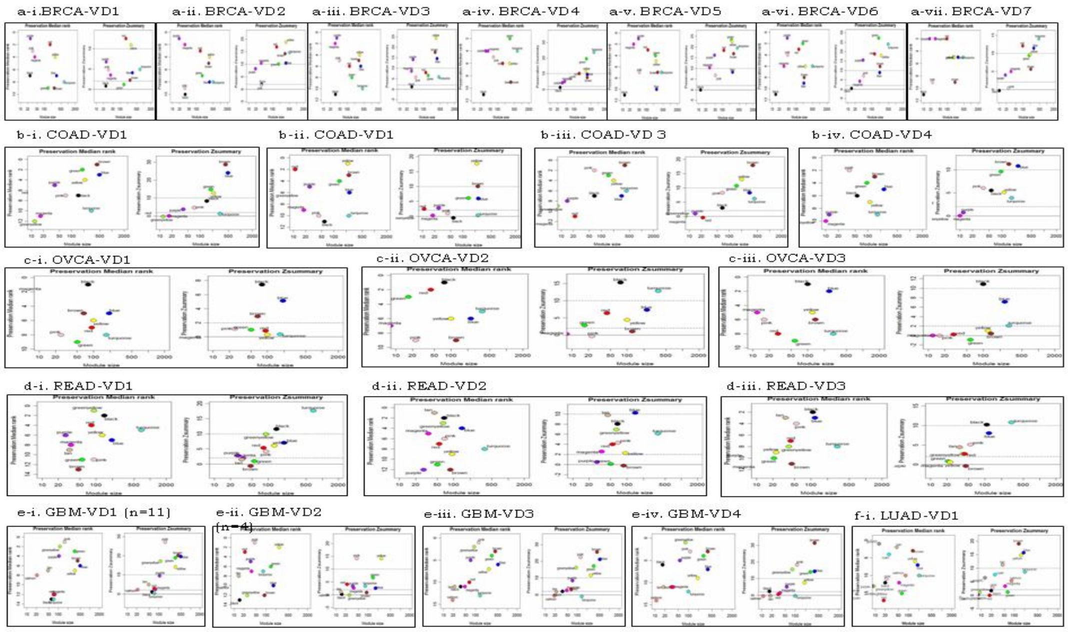

Supplementary Dataset3-Fig.2. Module preservation analysis (*medianRank* and *Zsummary* statistics) for cancer types across validation datasets, a-i-a-vii. BRCA, b-i-b-iv. COAD, c-i-c-iii. OVCA, d-i-d-iii. READ, e-i-e-iv. GBM, f-i. LUAD

Further module preservation statistics were computed as described in Expanded View Dataset2, and identified stability of co-expression relationships between genes of similar modules between the TCGA and validation data (UNC__AgilentG4502A_07 vs. Affymetrix; Supplementary Dataset3-Fig.2). This cross-platform validation identifies an overlap in expression and clustering of genes between the TCGA and validation dataset network modules (Supplementary Dataset3-Table2). However, of the three strongly conserved Gene Sets identified earlier (Supplementary Dataset2-Tables 2,3) only the Set1 (n=161) and Set2 (n=50) genes were at least moderately conserved in all validation datasets of the BRCA-COAD-OVCA-READ group, while the Set1-s genes were strongly conserved in the GBM-LUAD group (Supplementary Dataset3-Fig.3). Thereby, Set3 genes were discontinued from further analysis.

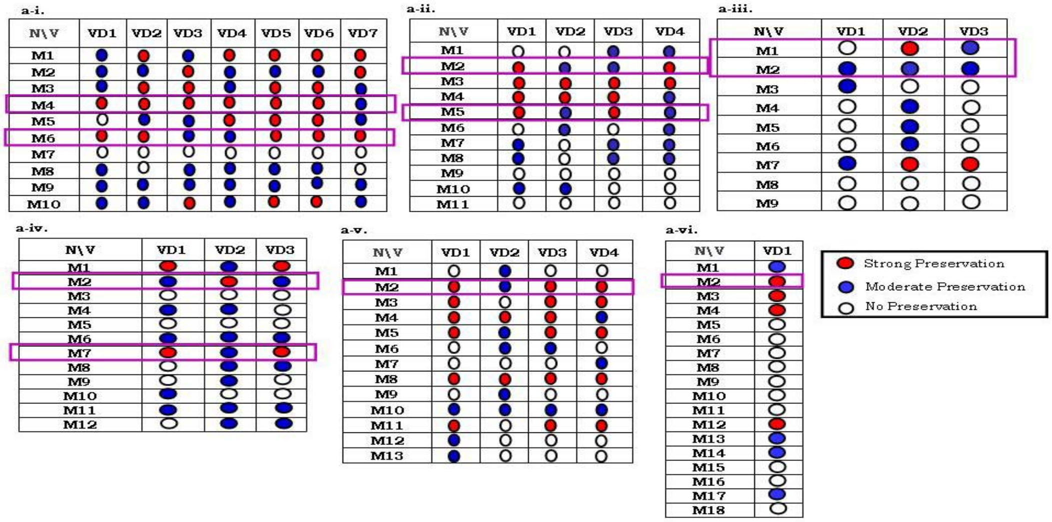

Supplementary Dataset3-Fig.3. Validation of WGCNA-based network modules in datasets downloaded from GEO (Expanded View Dataset3-Table1) for - a-i. BRCA, a-ii. COAD, a-iii.

OVCA, a-iv. READ, a-v. GBM and, a-vi. LUAD. Modules highlighted in the box represent Set 1 and Set 2 genes in case of the strongly preserved group (BRCA-COAD-OVCA-READ) and Set 1 genes for the moderately preserved group (GBM-LUAD).

References –

BRCA-VD1-VD7

1. Planche A, Bacac M, Provero P, Fusco C, Delorenzi M, Stehle JC, et al. Identification of prognostic molecular features in the reactive stroma of human breast and prostate cancer. PLoS.One 2011;6:e18640.
2. Iwamoto T, Bianchini G, Booser D, Qi Y, Coutant C, Shiang CY, Santarpia L, et al. Gene pathways associated with prognosis and chemotherapy sensitivity in molecular subtypes of breast cancer. J.Natl.Cancer Inst 2011;103:264-272.
3. Kretschmer C, Sterner-Kock A, Siedentopf F, Schoenegg W, Schlag PM, Kemmner W. Identification of early molecular markers for breast cancer. Mol.Cancer 2011;10:15.
4. Boersma BJ, Reimers M, Yi M, Ludwig JA, Luke BT, Stephens RM, et al. A stromal gene signature associated with inflammatory breast cancer. Int.J.Cancer 2008;122:1324-1332.
5. Turashvili G, Bouchal J, Baumforth K, Wei W, Dziechciarkova M, Ehrmann J, et al. Novel markers for differentiation of lobular and ductal invasive breast carcinomas by laser microdissection and microarray analysis. BMC.Cancer 2007;7: 55.

COAD-VD1-VD4

1. Schlicker A, Beran G, Chresta CM, McWalter G, Pritchard A, Weston S, et al. Subtypes of primary colorectal tumors correlate with response to targeted treatment in colorectal cell lines. BMC.Med.Genomics 2012;5:66.
2. Pfister TD, Reinhold WC, Agama K, Gupta S, Khin SA, Kinders RJ, et al. Topoisomerase I levels in the NCI-60 cancer cell line panel determined by validated ELISA and microarray analysis and correlation with indenoisoquinoline sensitivity. Mol.Cancer Ther 2009;8:1878-1884.
3. Snipstad K, Fenton CG, Kjaeve J, Cui G, Anderssen E, Paulssen RH. New specific molecular targets for radio-chemotherapy of rectal cancer. Mol.Oncol 2010;4:52-64.
4. Auman JT, Church R, Lee SY, Watson MA, Fleshman JW, Mcleod HL. Celecoxib pre-treatment in human colorectal adenocarcinoma patients is associated with gene expression alterations suggestive of diminished cellular proliferation. Eur.J.Cancer 2008;44:1754-1760.

OVCA-VD1-VD3

1. Bowen NJ, Walker LD, Matyunina LV, Logani S, Totten KA, Benigno BB, et al. Gene expression profiling supports the hypothesis that human ovarian surface epithelia are multipotent and capable of serving as ovarian cancer initiating cells. BMC.Med.Genomics 2009;2:71.
2. Peters D, Freund J, Ochs RL. Genome-wide transcriptional analysis of carboplatin response in chemosensitive and chemoresistant ovarian cancer cells. Mol.Cancer Ther 2005;4:1605-1616.
3. Moreno CS, Matyunina L, Dickerson EB, Schubert N, Bowen NJ, Logani S, Benigno BB, et al. Evidence that p53-mediated cell-cycle-arrest inhibits chemotherapeutic treatment of ovarian carcinomas. PLoS.One 2007;2:e441..

READ-VD1-VD3

1. Snipstad K, Fenton CG, Kjaeve J, Cui G, Anderssen E, Paulssen RH. New specific molecular targets for radio-chemotherapy of rectal cancer. Mol.Oncol 2010;4:52-64.

2. Tsuji S, Midorikawa Y, Takahashi T, Yagi K, Takayama T, Yoshida K, et al. Potential responders to FOLFOX therapy for colorectal cancer by Random Forests analysis. Br.J.Cancer 2012;106:126-132.

GBM-VD1-VD4

1. Grzmil M, Morin P Jr, Lino MM, Merlo A, Frank S, Wang Y, et al. MAP kinase-interacting kinase 1 regulates SMAD2-dependent TGF-beta signaling pathway in human glioblastoma. Cancer Res 2011;71:2392-2402.
2. Schulte A, Günther HS, Phillips HS, Kemming D, Martens T, Kharbanda S, et al. A distinct subset of glioma cell lines with stem cell-like properties reflects the transcriptional phenotype of glioblastomas and overexpresses CXCR4 as therapeutic target. Glia 2011;59:590-602.
3. Freije WA, Castro-Vargas FE, Fang Z, Horvath S, Cloughesy T, Liau LM, et al. Gene expression profiling of gliomas strongly predicts survival. Cancer Res 2004;64:6503-6510.
4. Sun L, Lee J, Fine HA. Neuronally expressed stem cell factor induces neural stem cell migration to areas of brain injury. J.Clin.Invest, 2004;113:1364-1374.

LUAD-VD1

1. Landi MT, Dracheva T, Rotunno M, Figueroa JD, Liu H, Dasgupta A, et al. Gene expression signature of cigarette smoking and its role in lung adenocarcinoma development and survival. PLoS.One 2008;3:e1651.

Supplementary Dataset4: Identification of Master Regulator Networks

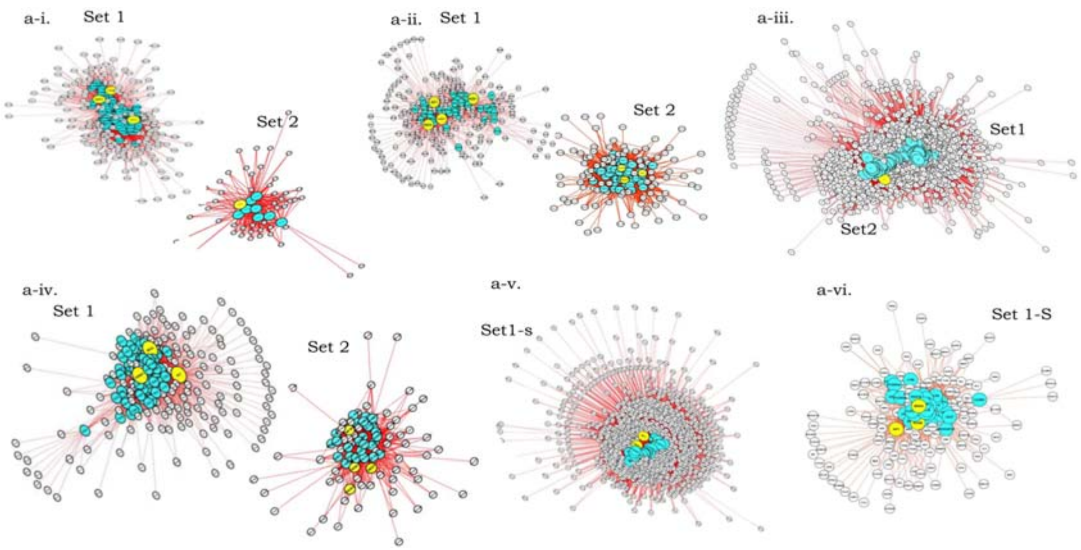

Supplementary Dataset4-Fig.1. Gene interaction networks for, a-i to a-iv. Set1 and Set2 genes in BRCA, COAD, OVCA, READ respectively; a-v, a-vi. Set1-s genes in GBM, LUAD respectively; Cyan colored nodes denote significant hubs (>= 10 interactions) while yellow colored nodes represent transcription factors.

Supplementary Dataset4-Table1: List of enriched hub markers in Supplementary Dataset4-Fig.1.

(i) BRCA-COAD-OVCA

BRCA			COAD		OVCA			
Set1		Set2	Set1	Set2	Set1			Set2
AIF1	CLEC4A	COL1A1	AIF1	PRRX1	AIF1	CD247	DPEP2	COL5A2
IKZF1	PTPRC	COL1A2	ITK	BGN	IKZF1	SIGLEC7	CD38	THBS2
ITK	APOBEC3H	COL5A1	ARHGAP30	COL10A1	TBX21	FCGR1A	JAK2	SPARC
ARHGAP30	CD5	COL5A2	ARHGAP9	COL11A1	MNDA	NPL	CD3D	COL5A1
ARHGAP9	CORO1A	COL6A3	BTK	COL12A1	SAMSN1	ICOS	VAV1	VCAN
BTK	CRTAM	ADAM12	HCLS1	COL1A1	LCP2	RNASE6	CORO1A	WISP1
HCLS1	CST7	CDH11	LCP2	COL1A2	CD53	SELPLG	LAG3	FAP
LCP2	EOMES	FAP	ARHGAP15	COL3A1	LAPTM5	NCF4	LRRC8C	COL6A3
PSCD4	GFI1		FYB	COL5A1	HAVCR2	TRPV2	KLHL6	INHBA
ARHGAP15	HAVCR2		CCR2	COL5A2	PLEK	SLAMF7	SUCNR1	COL1A2
FYB	IL2RB		CD2	COL6A3	PTPRC	CCR2	CD5	ITGA11
AMICA1	LCK		CD247	COL8A1	CYBB	EDG6	PVRIG	ADAM12
CCR2	LPXN		EVI2A	NID2	TAGAP	RAC2	APOBEC3H	CTSK
CCR7	LTB		EVI2B	POSTN	LAIR1	DOCK8	P2RY6	CDH11
CD2	NCKAP1L		HAVCR2	SPARC	TNFSF13B	PTAFR	APOBEC3G	COL3A1
CD247	PDCD1		HCST	ADAM12	CD48	IL7R	CD28	COL1A1
CD27	PIK3CG		ITGB2	CDH11	ARHGAP9	CD74	VMO1	PRRX1
CD28	PRKCB1		LAPTM5	CTHRC1	SLA	CCR1	CD3G	POSTN
EBI2	PSCDBP		P2RY10	CTSK	ITGB2	FCGR3A	IRAK3	COL11A1
EDG6	PSTPIP1		DOK3	FAP	FYB	ITGA4	PARVG	LOXL2
EVI2A	PTPN22		PLEK	INHBA	LILRB3	RGS1	LCP1	COL8A1
EVI2B	PVRIG		CYBB	ITGA11	CD2	CCR7	MMP9	P4HA3
HCST	RAC2		CCL5	ITGBL1	CCL5	IFI30	RASSF4	ITGBL1
ICOS	SH2D1A		CD33	LOXL2	PSCD4	CD52	LTB	LRRC15
IL21R	SIT1		CD37	MXRA5	URP2	P2RY10	HPSE	EDNRA
IL7R	SLAMF7		CD3D	NOX4	RGS18	VNN2	IGHM	SPON2
ITGB2	TBC1D10C		CD3G	P4HA3	TRAF3IP3	ITK	CCL19	PPEF1

KLHL6			CD48	CHSY-2	CD37	CST7	PSTPIP1	CTHRC1
LAPTM5			CD52	DIO2	NCF2	HCLS1	PDCD1	BGN
LILRB1			CD53	F2R	BTK	CD69	IGKC	MMP11
P2RY10			CD74	LRRC15	NCKAP1L	IL21R	ICAM1	RCN3
PARVG			CD96	MMP11	IL2RB	SH2D1A	CTSL1	TMEM46
DOK3			CLEC4A	PPAPDC1A	CD33	CD86	ACP5	COL10A1
PLEK			PTPRC	PPEF1	LILRB1	CD72	CCL17	NID2
CYBB			KLHL6	PRR16	PSCDBP	SIGLEC5	CHST11	TNFSF4
CCL17			IKZF1	RCN3	ARHGAP15	LPXN	GFI1	CORIN
CCL5			MNDA	SGIP1	EBI2	TRAT1	IL4I1	F2R
CD33			SAMSN1	SPARC	WDFY4	SLC37A2	JAKMIP1	DIO2
CD37			SLA	SPOCD1	ARHGDIB	CRTAM	PRKCB1	SGIP1
CD38			CCR1	SPON2	EVI2B	SIT1	LCK	COL12A1
CD3D			CD28	THBS2	ARHGAP30	PIK3CG	CLEC12A	MXRA5
CD3G			FCGR2B	THY1	FCGR2B	ADAM8	CD40	SPOCD1
CD48			LAIR1	TNFSF4	DOK3	PKD2L1	CLC	PRR16
CD52			LCP1	VCAN	EVI2A	CD27	TMEM149	THY1
CD53			CD86	WISP1	AMICA1	CD96	RAB42	NOX4
CD69			CTSL1	ZNF469	CLEC4A	KLHDC7B	PLA2G7	PPAPDC1A
CD72			IL2RB		PTPN22	HCST	TBC1D10C	
CD74					LY86	KLRC1	SLFN11	
CD96					TLR1	EOMES		

(i) READ-GBM-LUAD

**READ**			**GBM**		**LUAD**	
Set1		Set2	Set1-s		Set1-s	
AIF1	ITGA4	PRRX1	AIF1	LCP1	AIF1	LY86
MNDA	ITGB2	BGN	APOBEC3G	LCP2	ARHGAP30	MNDA
SAMSN1	KLHL6	COL10A1	ARHGAP30	LILRB1	ARHGAP9	NCF2
ITK	LAIR1	COL11A1	ARHGAP9	LPXN	BTK	NCKAP1L
ARHGAP30	LAPTM5	COL12A1	BTK	LY86	CD33	PLEK
ARHGAP9	LILRB1	COL1A1	CD33	MNDA	CD37	PTPRC
BTK	LILRB3	COL1A2	CD37	NCF2	CD53	RGS18
HCLS1	LY86	COL3A1	CD53	NCKAP1L	CD86	
LCP2	P2RY10	COL5A1	CD69	NPL	CLEC4A	
NCF2	PARVG	COL5A2	CD86	P2RY6	CYBB	
PSCD4	RGS18	COL6A3	CLEC4A	PLEK	EBI2	
ARHGAP15	SIGLEC7	COL8A1	CYBB	PSCDBP	EVI2B	
FYB	DOK3	NID2	EBI2	PTAFR	FCGR1A	
APOBEC3G	LCP1	POSTN	EVI2B	PTPRC	FCGR2B	
SLA	ACP5	SPARC	FCGR1A	RAB42	FCGR3A	
AMICA1	PLEK	ADAM12	FCGR2B	RGS18	FYB	
CCR1	CYBB	CDH11	FCGR3A	RNASE6	HAVCR2	
CCR2	CCL17	CTHRC1	FYB	SAMSN1	HCLS1	
CCR7	CCL5	CTSK	HAVCR2	SIGLEC5	IFI30	
CD2	PLA2G7	FAP	HCLS1	SIGLEC7	IL4I1	
CD247	NCKAP1L	INHBA	IFI30	SLA	LAIR1	
CD27	CD33	ITGA11	IL4I1	SLC37A2	LAPTM5	
CD28	CD37	ITGBL1	ITGB2	SUCNR1	LCP1	
DPEP2	CD38	LOXL2	KLHDC7B	TLR1	LCP2	
EBI2	CD3D	MXRA5	LAIR1	VMO1	LILRB1	
EDG6	CD3G	NOX4	LAPTM5	VNN2	LPXN	
EVI2A	CD48	P4HA3				
EVI2B	CD52					
FCGR1A	CD53					
FCGR2B	CD69					
HAVCR2	CD72					
HCST	CD74					
ICOS	CD86					

IFI30	CD96					
IL21R	CLEC4A					
IL7R	PTPRC					

Supplementary Dataset4-Table2: Transcription factors within interaction network of Set1 (BRCA-COAD-OVCA-READ) and Set1-s (GBM-LUAD), red color denotes significant hub markers while blue color denotes interacting partners

Set1						Set2			
**BRCA**	**COAD**	**OVCA**	**READ**	**GBM**	**LUAD**	**BRCA**	**COAD**	**OVCA**	**READ**
AIF1	AIF1	AIF1	AIF1	AIF1	AIF1	PRRX1	PRRX1	PRRX1	PRRX1
IKZF1	IKZF1	IKZF1	IKZF1	IKZF1	-	-	TWIST1	TWIST1	TWIST1
MNDA	MNDA	MNDA	MNDA	MNDA	MNDA	-	-	SNAI2	SNAI2
SAMSN1	SAMSN1	SAMSN1	SAMSN1	SAMSN1	SAMSN1	-	-	ZEB1	-
EOMES	-	EOMES	-	-	-	-	-	ZEB2	-
GFI1	-	GFI1	-	GFI1	-	-	-	SNAI1	-
-	-	KLHDC7B	-	KLHDC7B	KLHDC7B	-	-	-	-
EVI2A	EVI2A	EVI2A	EVI2A	EVI2A	EVI2A	-	-	-	-
EVI2B	EVI2B	EVI2B	EVI2B	EVI2B	EVI2B	-	-	-	-
TBX21	TBX21	TBX21	TBX21	TBX21	-	-	-	-	-

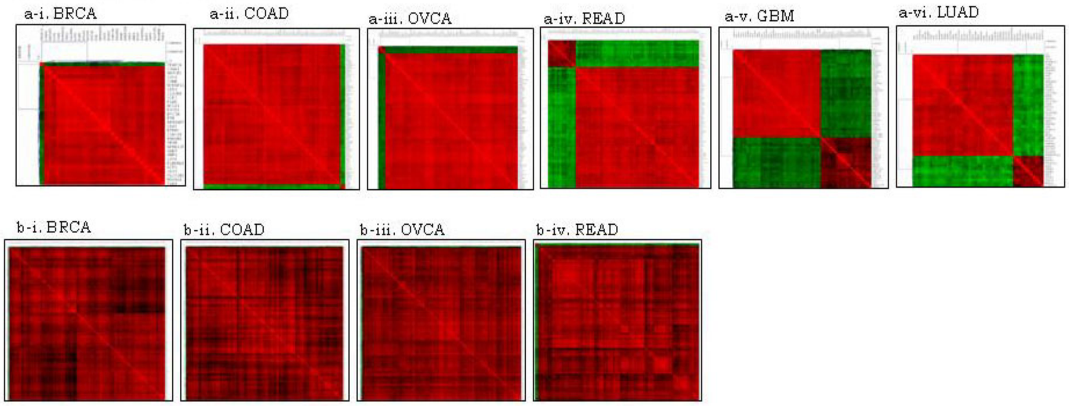

Supplementary Dataset4-Fig.2. Correlation analysis for AIF1 and PRRX1 expression within six cancer types. a-i-a-vi . Correlation plot for genes correlated with AIF1 for BRCA, COAD, OVCA, READ, GBM and LUAD cancer types respectively. b-i-b-iv. Correlation plot for genes correlated with PRRX1 for BRCA, COAD, OVCA, READ, GBM and LUAD cancer types respectively. Correlation value denoted with red color shows positive correlation while green colored denotes negative correlation among genes.

Supplementary Dataset4-Table3: Identification of classifiers for 3 sets

SET1 classifiers(36)	SET2 classifiers(8)	Set1-s classifiers (33)
AIF1	COL1A1	AIF1
ITK	COL1A2	ARHGAP30
ARHGAP30	COL5A1	ARHGAP9
ARHGAP9	COL5A2	BTK
BTK	COL6A3	CD33
HCLS1	ADAM12	CD37
LCP2	CDH11	CD53
ARHGAP15	FAP	CD86
FYB		CLEC4A
CCR2		CYBB
CD2		EBI2
CD247		EVI2B

CD28		FCGR1A
EVI2A		FCGR2B
EVI2B		FCGR3A
HCST		FYB
ITGB2		HAVCR2
KLHL6		HCLS1
LAPTM5		IFI30
P2RY10		IL4I1
DOK3		LAIR1
PLEK		LAPTM5
CYBB		LCP1
CCL5		LCP2
CD33		LILRB1
CD37		LPXN
CD3D		LY86
CD3G		MNDA
CD48		NCF2
CD52		NCKAP1L
CD53		PLEK
CD74		PTPRC
CD96		RGS18
CLEC4A		
PTPRC		
HAVCR2		

Supplementary Dataset5: Validation of AIF1 and PRRX1 as master regulators independent of TCGA in validation datasets of 6 cancers

AIF1 and PRRX1 were predicted as master gene regulators of immunomodulation and metastases functionalities. To confirm that these results were not specific to the TCGA gene expression datasets, we identified the weighted gene interactions regulated by Set1/Set1-s and Set2 genes independently in other datasets of six cancers (Supplementary Dataset3-Table1). Towards this purpose, validation datasets which showed strongest preservation (in context of *Zsummary* and *medianRank* statistics) of Set1/Set1-s and Set2 for six cancers were selected for network analysis and visualization (Supplementary Dataset3.Fig.3). Comparing gene interaction network outputs between the TCGA and these select validation dataset analyses identified similar partners across these datasets and thereby affirmed regulation of genes and TFs involved in immune responses and metastasis to be governed by AIF1 and PRRX1 respectively (Supplementary Dataset5-Fig.1; Supplementary Dataset5-Tables 1,2).

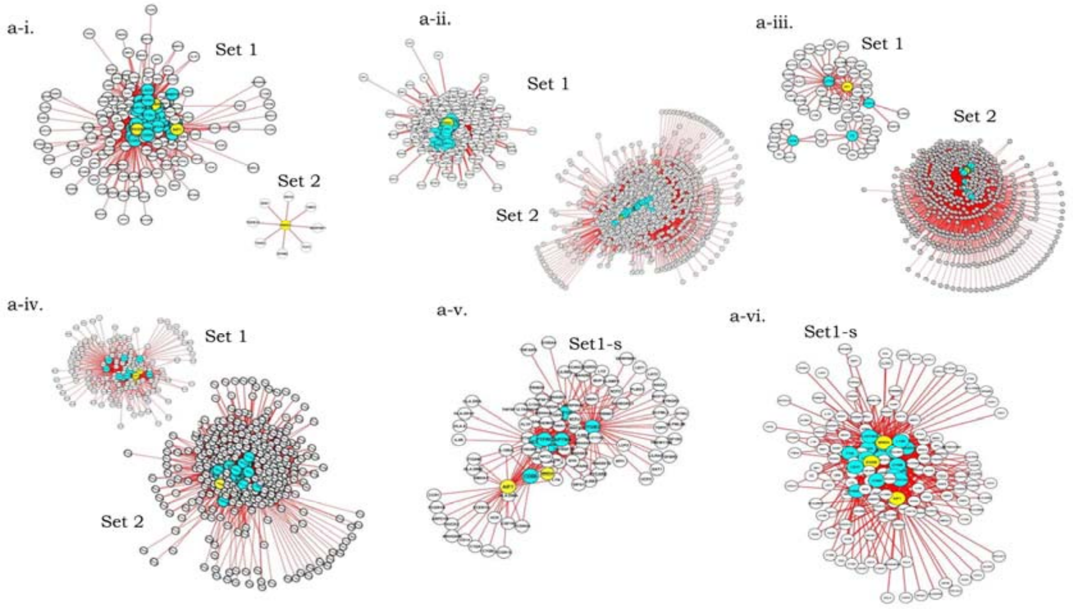

Supplementary Dataset5-Fig.1.a-i to a-iv. Gene interaction networks for Set1 and Set2 genes in BRCA, COAD, OVCA, READ validation datasets respectively; a-v, a-vi. Set1-s genes in GBM, LUAD validation datasets respectively; Cyan colored nodes denote significant hubs (>= 10 interactions) while yellow colored nodes represent transcription factors.

Supplementary Dataset5-Table1. Common interactors of AIF1 between TCGA and validation datasets

BRCA - VD3	BRCA - VD5	BRCA - VD6	COAD - VD1	COAD - VD4	OVCA - VD1	READ - VD2	GBM -VD1			GBM - VD3	GBM - VD1
**2**	**20**	**21**	**44**	**7**	**16**	**11**	**106**			**27**	**37**
CD53	C3AR1	C3AR1	ALOX5AP	CD14	C1QB	ALOX5AP	ALOX5	GIMAP6	RASSF5	ARHGDIB	ALOX5AP
NCF4	CD53	CD53	AMICA1	CLEC2B	CD14	CCL2	AOAH	GIMAP8	RGS18	C1QA	BCL2A1
	DOCK2	CLEC4A	ARHGAP9	DPYD	CD163	CD48	APBB1IP	GPR34	RNASE6	C1QB	C3AR1
	EVI2A	CYBB	CCR1	FCER1G	CD48	CD52	APOC2	HAVCR2	SAMSN1	CCR1	CCL2
	EVI2B	DOCK2	CCR2	LAPTM5	CD53	CD72	ARHGAP15	HCK	SCIN	CD14	CD52
	FYB	EVI2B	CD14	PLA2G7	CD68	CD74	ARHGAP30	HCLS1	SIGLEC10	CSF1R	CD53
	HCLS1	FYB	CD300A	VAMP5	EVI2B	CD86	ARHGAP9	HLA.DMA	SLA	DOCK2	CD86
	IL10RA	HCLS1	CD300LF		HCLS1	HAVCR2	BLNK	HLA.DMB	SLC7A7	FCER1G	CLEC4A
	LAIR1	IL10RA	CD48		HLA.DMB	HLA.DRA	BST2	HLA.DOA	SLCO2B1	FCGR2A	CYBB
	LAPTM5	LAPTM5	CD52		HLA.DRA	LST1	C1QA	HLA.DPA1	TFEC	HCK	EVI2A
	LCP2	LCP2	CD53		ITGB2	RGS1	C1QB	HLA.DPB1	TLR1	HCLS1	EVI2B
	LY86	LY86	CD69		MYO5A		C1QC	HLA.DRA	TLR2	HLA.DMA	FCER1G

	MNDA	MNDA	CD86		NCF4		C3	HLA.DRB5	TLR7	HLA.DMB	FCGR2A
	NCF4	NCF4	CLEC2B		NCKAP1L		C3AR1	HLA.E	TNFRSF1B	HMHA1	FCGR2B
	PLEK	PLEK	CLEC4A		SLA		CASP1	HMHA1	TNFSF13B	ITGAM	FYB
	PTPRC	PTPRC	DOK3		SRGN		CCR1	IGSF6	TREM2	ITGB2	GMFG
	RNASE6	RNASE6	EVI2A				CD14	IKZF1	TYROBP	LAIR1	HCK
	SAMSN1	SAMSN1	EVI2B				CD163	IL6R	VSIG4	LAPTM5	HLA.DPA1
	SELPLG	SELPLG	FLI1				CD53	IRF8		LY96	HLA.DRA
	SLCO2B1	SLCO2B1	GAB3				CD74	ITGB2		MNDA	IFI30
		TRPV2	HAVCR2				CD84	LAIR1		PTPRC	ITGAM
			HCLS1				CD86	LAPTM5		SAMSN1	ITGB2
			HCST				CLEC7A	LAT2		SLA	LAPTM5
			HLA.DMB				CSF1R	LCP2		STAB1	LCP1
			ITGA4				CSTA	LILRA2		TLR2	LCP2
			ITGB2				CTSH	LILRB1		TYROBP	LY86
			LILRB1				CTSS	LY86		VSIG4	MAFB
			LST1				CXCL16	LYZ			MNDA
			LY86				CYBB	MAFB			MS4A4A
			LY96				CYSLTR1	MGAT4A			MS4A6A
			MS4A4A				DOCK2	MNDA			MSR1
			MS4A6A				DOCK8	MPEG1			PLEK
			NCF2				EVI2B	MS4A4A			PTPRC
			NCKAP1L				FCER1G	MS4A6A			RNASE6
			PLEK				FCGBP	MS4A7			SLCO2B1
			RHOH				FCGR2A	MSR1			SNX10
			SAMSN1				FGL2	MYO1F			TFEC
			SLA				FKBP5	NCF4			
			SLAMF7				FPR1	NPL			
			SRGN				FYB	PARVG			
			STX11				GIMAP1	PIK3AP1			
			TRAF3IP3				GIMAP2	PLEK			
			TRPV2				GIMAP4	PSMB9			
			WIPF1				GIMAP5	PTPRC			

Supplementary Dataset4-Table2. Common interactors of PRRX1 from TCGA and GEO datasets

COAD	OVCA		READ
**TCGA-VD1**	**TCGA-VD1**	**TCGA-VD2**	**TCGA-VD1**
**10**	**2**	**13**	**5**
COL15A1	TCEAL7	RND3	RAB31
FSTL3	VIM	SCG2	SPARC
ITGA5		SEC24D	SULF1
LEPRE1		SLC2A3	THBS2
PDPN		SPARC	VCAN
SGIP1		SPOCK1	
SPARC		SRPX	
SPOCK1		TIMP2	
SPON2		TIMP3	
WISP1		TMEM158	
		TMEM47	
		TPM1	
		TPM2	

Supplementary Dataset6: Identification of pathways associated with cancers

Pathways regulated within classes were identified using expression of Set1 (n=161) and Set2 (n=50) genes for BRCA-COAD-OVCA-READ cancers. Set1-s genes were used to identify pathways regulated within classes of GBM-LUAD cancer types using Gene Set Enrichment Analysis (GSEA) software. Cytoscape 2.8.3 was used to generate pathway network using enrichment map plugin ($p<0.05$). Pathways regulated by Set1 in 4 cancers were up-regulated for class 1 and class 2 whereas for class 3 and 4 it shows downregulation (Supplementary Dataset6-Fig 1; Supplementary Dataset6-Table 1); Two subtypes of GBM-LUAD show up and down-regulation of pathways which were regulated by Set1-s genes (Supplementary Dataset6-Fig 2; Supplementary Dataset6-Table 2) and involved in immune functionalities. Set2 genes were responsible for up-regulation of pathways within class 1 and 3 patients and down-regulation of class 2 and 4 patients (Supplementary Dataset6-Fig 3; Supplementary Dataset6-Table 3).

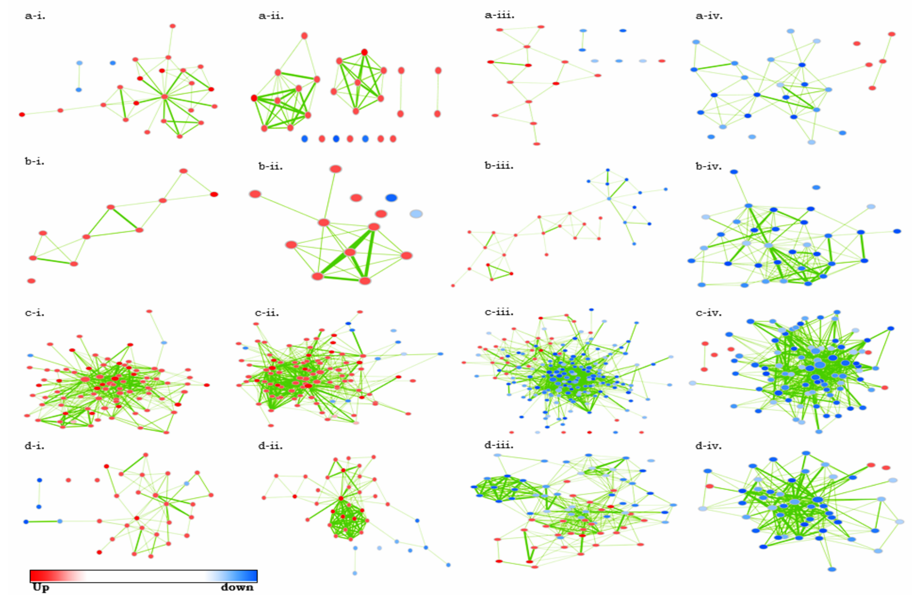

Supplementary Dataset6-Fig.1. GSEA based pathway analysis for Set1 genes visualized as network ($p<0.05$) for classes of BRCA, COAD, OVCA, READ (a-d respectively); shared genes are indicated by connecting lines where thickness of line denotes number of shared genes.

Supplementary Dataset6-Table1. Set1 associated Pathways within 4 classes of BRCA-COAD-OVCA-READ

NO.	Class 1	Class 2	Class 3	Class 4
		**BRCA**		
1	HAN_SATB1_TARGETS_UP	PID_SHP2_PATHWAY	CROONQUIST_IL6_DEPRIVATION_UP	PANGAS_TUMOR_SUPPRESSION_BY_SMAD1_AND_SMAD5_UP
2	LEE_DIFFERENTIATING_T_LYMPHOCYTE	REACTOME_PHOSPHORYLATION_OF_CD3_AND_TCR_ZETA_CHAINS	BIOCARTA_TALL1_PATHWAY	
3	MORI_SMALL_PRE_BII_LYMPHOCYTE_UP	BIOCARTA_IL2_PATHWAY		
4		GOLDRATH_IMMUNE_MEMORY		
5		GAVIN_FOXP3_TARGETS_CLUSTER_P2		
6		PLASARI_TGFB1_SIGNALING_VIA_NFIC_10HR_UP		
7		MARZEC_IL2_SIGNALING_UP		
8		PID_IL2_1PATHWAY		
9		GAVIN_IL2_RESPONSIVE_FOXP3_TARGETS_UP		
10		PID_IL2_STAT5PATHWAY		

11		BIOCARTA_TCRA_PATHWAY		
12		BIOCARTA_CTL_PATHWAY		
13		BIOCARTA_IL17_PATHWAY		
**COAD**				
1	LIAN_NEUTROPHIL_GRANULE_CONSTITUENTS	ST_B_CELL_ANTIGEN_RECEPTOR	SANA_TNF_SIGNALING_UP	WESTON_VEGFA_TARGETS
2	TONKS_TARGETS_OF_RUNX1_RUNX1T1_FUSION_ERYTHROCYTE_UP	REACTOME_ANTIGEN_ACTIVATES_B_CELL_RECEPTOR_LEADING_TO_GENERATION_OF_SECOND_MESSENGERS	LABBE_TARGETS_OF_TGFB1_AND_WNT3A_DN	WESTON_VEGFA_TARGETS_3HR
3		REACTOME_SIGNALING_BY_THE_B_CELL_RECEPTOR_BCR	GROSS_HIF1A_TARGETS_UP	ZHENG_FOXP3_TARGETS_IN_T_LYMPHOCYTE_DN
4		ST_FAS_SIGNALING_PATHWAY	HAMAI_APOPTOSIS_VIA_TRAIL_DN	KEGG_FC_GAMMA_R_MEDIATED_PHAGOCYTOSIS
5		ZHOU_INFLAMMATORY_RESPONSE_LIVE_UP	PID_INTEGRIN1_PATHWAY	
6			SOUCEK_MYC_TARGETS	
7			GAVIN_FOXP3_TARGETS_CLUSTER_P7	
8			DEURIG_T_CELL_PROLYMPHOCYTIC_LEUKEMIA_DN	
**OVCA**				
1	BIOCARTA_GSK3_PATHWAY	POOLA_INVASIVE_BREAST_CANCER_UP	WINTER_HYPOXIA_UP	SANSOM_APC_TARGETS_UP
2	WINTER_HYPOXIA_DN	REACTOME_IMMUNE_SYSTEM	MARSON_BOUND_BY_FOXP3_STIMULATED	NAKAJIMA_MAST_CELL
3	SCHUETZ_BREAST_CANCER_DUCTAL_INVASIVE_UP	BIOCARTA_IL12_PATHWAY	REACTOME_IMMUNE_SYSTEM	WATANABE_ULCERATIVE_COLITIS_WITH_CANCER_DN
4	KASLER_HDAC7_TARGETS_2_UP	JAZAG_TGFB1_SIGNALING_VIA_SMAD4_UP	BROWN_MYELOID_CELL_DEVELOPMENT_DN	THEILGAARD_NEUTROPHIL_AT_SKIN_WOUND_UP
5	ACOSTA_PROLIFERATION_INDEPENDENT_MYC_TARGETS_DN	REACTOME_ADAPTIVE_IMMUNE_SYSTEM	REACTOME_COSTIMULATION_BY_THE_CD28_FAMILY	HADDAD_T_LYMPHOCYTE_AND_NK_PROGENITOR_DN
6	PEDERSEN_METASTASIS_BY_ERBB2_ISOFORM_6	BIOCARTA_DC_PATHWAY	MARSON_BOUND_BY_FOXP3_UNSTIMULATED	TONKS_TARGETS_OF_RUNX1_RUNX1T1_FUSION_HSC_DN
7	GILDEA_METASTASIS	GAVIN_PDE3B_TARGETS	REACTOME_ADAPTIVE_IMMUNE_SYSTEM	WIERENGA_STAT5A_TARGETS_GROUP2
8	WORSCHECH_TUMOR_EVASION_AND_TOLEROGENICITY_UP	BIOCARTA_TCYTOTOXIC_PATHWAY	SIG_CHEMOTAXIS	REACTOME_SEMA4D_IN_SEMAPHORIN_SIGNALING
9	THEILGAARD_NEUTROPHIL_AT_SKIN_WOUND_UP	BIOCARTA_THELPER_PATHWAY	BIOCARTA_TCYTOTOXIC_PATHWAY	HAN_JNK_SINGALING_UP
10	RAMALHO_STEMNESS_DN	BIOCARTA_IL17_PATHWAY	BIOCARTA_THELPER_PATHWAY	XU_HGF_TARGETS_REPRESSED_BY_AKT1_DN
11	HAHTOLA_CTCL_PATHOGENESIS	LEE_DIFFERENTIATING_T_LYMPHOCYTE	BIOCARTA_TCR_PATHWAY	BIOCARTA_TOLL_PATHWAY
12	LINDSTEDT_DENDRITIC_CELL_MATURATION_D	NGUYEN_NOTCH1_TARGETS_DN	BASSO_CD40_SIGNALING_DN	SANSOM_APC_TARGETS_DN
13	BROWN_MYELOID_CELL_DEVELOPMENT_UP	KONDO_HYPOXIA	WANG_RESPONSE_TO_GSK3_INHIBITOR_SB216763_UP	BIOCARTA_GSK3_PATHWAY
14	YE_METASTATIC_LIVER_CANCER	FAELT_B_CLL_WITH_VH_REARRANGEMENTS_UP	KEGG_T_CELL_RECEPTOR_SIGNALING_PATHWAY	PID_INTEGRIN_CS_PATHWAY
15	HOEBEKE_LYMPHOID_STEM_CELL_UP	THEILGAARD_NEUTROPHIL_AT_SKIN_WOUND_UP	HADDAD_T_LYMPHOCYTE_AND_NK_PROGENITOR_DN	GAVIN_PDE3B_TARGETS
16	KEGG_CELL_ADHESION_MOLECULES_CAMS	REACTOME_IL_RECEPTOR_SHC_SIGNALING	CHIARETTI_T_ALL_REFRACTORY_TO_THERAPY	PID_INTEGRIN1_PATHWAY
17	BIOCARTA_NKT_PATHWAY	WANG_METASTASIS_OF_BREAST_CANCER_ESR1_DN	REACTOME_TCR_SIGNALING	SWEET_KRAS_TARGETS_UP
18		GAVIN_FOXP3_TARGETS_CLUSTER_T4	ONO_FOXP3_TARGETS_UP	MORI_PRE_BI_LYMPHOCYTE_UP
19		PEDERSEN_METASTASIS_BY_ERBB2_ISOFORM_6	KONDO_HYPOXIA	PID_FCER1PATHWAY
20		WINTER_HYPOXIA_UP	BIOCARTA_CTLA4_PATHWAY	GAVIN_FOXP3_TARGETS_CLUSTER_P4
21			SIG_BCR_SIGNALING_PATHWAY	YE_METASTATIC_LIVER_CANCER
22			KEGG_CHEMOKINE_SIGNALING_PATHWAY	POOLA_INVASIVE_BREAST_CANCER_UP
23			LIAO_HAVE_SOX4_BINDING_SITES	PEDERSEN_METASTASIS_BY_ERBB2_ISOFORM_6
24			DEURIG_T_CELL_PROLYMPHOCYTI	GILDEA_METASTASIS

			C_LEUKEMIA_DN	
25			LEE_DIFFERENTIATING_T_LYMPHOCYTE	REACTOME_TRAF6_MEDIATED_IRF7_ACTIVATION_IN_TLR7_8_OR_9_SIGNALING
26			REACTOME_PHOSPHORYLATION_OF_CD3_AND_TCR_ZETA_CHAINS	HAHTOLA_CTCL_PATHOGENESIS
27			BIOCARTA_TCRA_PATHWAY	KASLER_HDAC7_TARGETS_2_UP
28			BIOCARTA_BCR_PATHWAY	BROWN_MYELOID_CELL_DEVELOPMENT_UP
29			REACTOME_DOWNSTREAM_TCR_SIGNALING	
30			PID_CD8TCRPATHWAY	
31			SIG_PIP3_SIGNALING_IN_B_LYMPHOCYTES	
32			PID_TCR_PATHWAY	
33			HOLLMANN_APOPTOSIS_VIA_CD40_UP	
34			REACTOME_INNATE_IMMUNE_SYSTEM	
35			BIOCARTA_DC_PATHWAY	
36			REACTOME_G_ALPHA_I_SIGNALLING_EVENTS	
37			KEGG_B_CELL_RECEPTOR_SIGNALING_PATHWAY	
38			PILON_KLF1_TARGETS_DN	
39			REACTOME_TOLL_RECEPTOR_CASCADES	
40			ZHENG_FOXP3_TARGETS_IN_T_LYMPHOCYTE_DN	
41			MATSUDA_NATURAL_KILLER_DIFFERENTIATION	
42			THEILGAARD_NEUTROPHIL_AT_SKIN_WOUND_UP	
43			LI_INDUCED_T_TO_NATURAL_KILLER_DN	
44			BIOCARTA_NKT_PATHWAY	
45			MARSON_FOXP3_TARGETS_STIMULATED_UP	
46			PID_BCR_5PATHWAY	
47			PID_FCER1PATHWAY	
48			BOSCO_TH1_CYTOTOXIC_MODULE	
49			SHIN_B_CELL_LYMPHOMA_CLUSTER_3	
**READ**				
1	BIOCARTA_BLYMPHOCYTE_PATHWAY	LEE_DIFFERENTIATING_T_LYMPHOCYTE	SWEET_KRAS_TARGETS_UP	BIOCARTA_BLYMPHOCYTE_PATHWAY
2	PID_INTEGRIN2_PATHWAY	BIOCARTA_TCRA_PATHWAY	PID_INTEGRIN1_PATHWAY	COATES_MACROPHAGE_M1_VS_M2_UP
3	MARSON_FOXP3_TARGETS_STIMULATED_UP	REACTOME_PHOSPHORYLATION_OF_CD3_AND_TCR_ZETA_CHAINS	MANTOVANI_NFKB_TARGETS_UP	ZHAN_MULTIPLE_MYELOMA_CD1_AND_CD2_UP
4	PID_NFAT_TFPATHWAY	REACTOME_TCR_SIGNALING	MANTOVANI_VIRAL_GPCR_SIGNALING_UP	ZAMORA_NOS2_TARGETS_DN
5		REACTOME_DOWNSTREAM_TCR_SIGNALING	PID_INTEGRIN2_PATHWAY	PANGAS_TUMOR_SUPPRESSION_BY_SMAD1_AND_SMAD5_UP
6		PURBEY_TARGETS_OF_CTBP1_NOT_SATB1_DN	MARTINELLI_IMMATURE_NEUTROPHIL_DN	HUANG_GATA2_TARGETS_UP
7		BIOCARTA_IL12_PATHWAY	WESTON_VEGFA_TARGETS_6HR	PID_INTEGRIN_CS_PATHWAY
8		REACTOME_TRANSLOCATION_OF_ZAP_70_TO_IMMUNOLOGICAL_SYNAPSE	PID_INTEGRIN5_PATHWAY	PLASARI_TGFB1_SIGNALING_VIA_NFIC_1HR_UP
9		REACTOME_PD1_SIGNALING	ZHOU_TNF_SIGNALING_4HR	
10		ALCALA_APOPTOSIS	KOINUMA_TARGETS_OF_SMAD2_OR_SMAD3	
11		KEGG_NEUROACTIVE_LIGAND_RECEPTOR_INTERACTION	PLASARI_TGFB1_SIGNALING_VIA_NFIC_10HR_UP	
12		CROONQUIST_IL6_DEPRIVATION_DN	BIOCARTA_IL12_PATHWAY	
13		HAMAI_APOPTOSIS_VIA_TRAIL_UP	REACTOME_TRANSLOCATION_OF_ZAP_70_TO_IMMUNOLOGICAL_SYNAPSE	

14		PID_CXCR4_PATHWAY	REACTOME_PD1_SIGNALING	
15		PID_INTEGRIN_A4B1_PATHWAY	WUNDER_INFLAMMATORY_RESPONSE_AND_CHOLESTEROL_UP	
16		HOFFMANN_SMALL_PRE_BII_TO_IMMATURE_B_LYMPHOCYTE_DN	REACTOME_NFKB_AND_MAP_KINASES_ACTIVATION_MEDIATED_BY_TLR4_SIGNALING_REPERTOIRE	
17			BIOCARTA_CTL_PATHWAY	
18			BIOCARTA_TALL1_PATHWAY	
19			REACTOME_G_ALPHA_I_SIGNALLING_EVENTS	
20			PURBEY_TARGETS_OF_CTBP1_NOT_SATB1_DN	
21			BIOCARTA_TCR_PATHWAY	
22			LI_INDUCED_T_TO_NATURAL_KILLER_UP	
23			MARSON_BOUND_BY_FOXP3_UNSTIMULATED	
24			HUTTMANN_B_CLL_POOR_SURVIVAL_UP	
25			REACTOME_GPCR_LIGAND_BINDING	
26			REACTOME_IMMUNOREGULATORY_INTERACTIONS_BETWEEN_A_LYMPHOID_AND_A_NON_LYMPHOID_CELL	
27			WANG_RESPONSE_TO_GSK3_INHIBITOR_SB216763_UP	
28			BIOCARTA_GSK3_PATHWAY	
29			REACTOME_ACTIVATION_OF_IRF3_IRF7_MEDIATED_BY_TBK1_IKK_EPSILON	

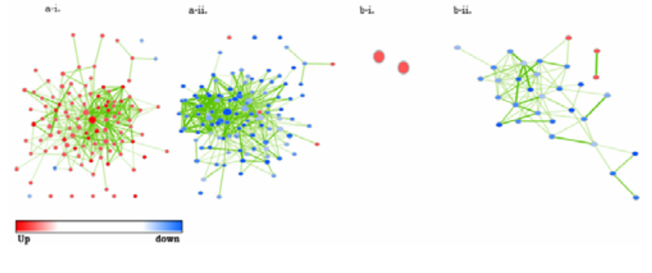

Supplementary Dataset6-Fig.2. GSEA based pathway analysis for Set1-s (n=55) visualized as network (p value<0.05) for two subtypes of (a) GBM and (b) LUAD.

Supplementary Dataset6-Table2. Set1-s associated pathways in 2 classes of GBM and LUAD

	GBM		LUAD
**NO.**	**Class 1**	**Class 2**	**Class 2**
1	KEGG_FC_GAMMA_R_MEDIATED_PHAGOCYTOSIS	KEGG_FC_GAMMA_R_MEDIATED_PHAGOCYTOSIS	BASSO_B_LYMPHOCYTE_NETWORK
2	WANG_RESPONSE_TO_GSK3_INHIBITOR_SB216763_UP	WANG_RESPONSE_TO_GSK3_INHIBITOR_SB216763_UP	PANGAS_TUMOR_SUPPRESSION_BY_SMAD1_AND_SMAD5_UP
3	SIG_PIP3_SIGNALING_IN_B_LYMPHOCYTES	SIG_PIP3_SIGNALING_IN_B_LYMPHOCYTES	MARZEC_IL2_SIGNALING_DN
4	ZHENG_FOXP3_TARGETS_IN_T_LYMPHOCYTE_DN	ZHENG_FOXP3_TARGETS_IN_T_LYMPHOCYTE_DN	HAMAI_APOPTOSIS_VIA_TRAIL_UP
5	ST_T_CELL_SIGNAL_TRANSDUCTION	RAY_TARGETS_OF_P210_BCR_ABL_FUSION_DN	KAMIKUBO_MYELOID_CEBPA_NETWORK
6	PID_PI3KCIPATHWAY	KEGG_FC_EPSILON_RI_SIGNALING_PATHWAY	
7	KEGG_B_CELL_RECEPTOR_SIGNALING_PATHWAY	MARTENS_BOUND_BY_PML_RARA_FUSION	
8	REACTOME_PLATELET_ACTIVATION_SIGNALING_AND_AGGREGATION	POOLA_INVASIVE_BREAST_CANCER_UP	
9	PILON_KLF1_TARGETS_DN	REACTOME_PLATELET_ACTIVATION_SIGNALING_AND_AGGREGATION	
10	PID_TCR_PATHWAY	OKAMOTO_LIVER_CANCER_MULTICENTRIC_OCCURRENCE_UP	
11	KEGG_FC_EPSILON_RI_SIGNALING_PA	REACTOME_SIGNALING_BY_RHO_GTPASES	

	THWAY		
12	REACTOME_ANTIGEN_ACTIVATES_B_CELL_RECEPTOR_LEADING_TO_GENERATION_OF_SECOND_MESSENGERS	ST_T_CELL_SIGNAL_TRANSDUCTION	
13	SIG_BCR_SIGNALING_PATHWAY	WIERENGA_STAT5A_TARGETS_DN	
14	REACTOME_TOLL_RECEPTOR_CASCADES	THEILGAARD_NEUTROPHIL_AT_SKIN_WOUND_DN	
15	KEGG_LEUKOCYTE_TRANSENDOTHELIAL_MIGRATION	SIG_BCR_SIGNALING_PATHWAY	
16	KEGG_ARACHIDONIC_ACID_METABOLISM	PILON_KLF1_TARGETS_DN	
17	THEILGAARD_NEUTROPHIL_AT_SKIN_WOUND_DN	KEGG_LEUKOCYTE_TRANSENDOTHELIAL_MIGRATION	
18	LEE_DIFFERENTIATING_T_LYMPHOCYTE	REACTOME_TOLL_RECEPTOR_CASCADES	
19	HASLINGER_B_CLL_WITH_13Q14_DELETION	REACTOME_TCR_SIGNALING	
20	YAGI_AML_FAB_MARKERS	REACTOME_ANTIGEN_ACTIVATES_B_CELL_RECEPTOR_LEADING_TO_GENERATION_OF_SECOND_MESSENGERS	
21	REACTOME_TCR_SIGNALING	LEE_DIFFERENTIATING_T_LYMPHOCYTE	
22	BOHN_PRIMARY_IMMUNODEFICIENCY_SYNDROM_DN	LU_IL4_SIGNALING	
23	REACTOME_ADAPTIVE_IMMUNE_SYSTEM	KAMIKUBO_MYELOID_CEBPA_NETWORK	
24	GAVIN_FOXP3_TARGETS_CLUSTER_T7	HOFFMANN_SMALL_PRE_BII_TO_IMMATURE_B_LYMPHOCYTE_DN	
25	ZHOU_INFLAMMATORY_RESPONSE_FIMA_DN	ZHOU_INFLAMMATORY_RESPONSE_LIVE_DN	
26	REACTOME_REGULATION_OF_SIGNALING_BY_CBL	BOHN_PRIMARY_IMMUNODEFICIENCY_SYNDROM_DN	
27		GAVIN_FOXP3_TARGETS_CLUSTER_T7	
28		MORI_LARGE_PRE_BII_LYMPHOCYTE_DN	
29		HADDAD_T_LYMPHOCYTE_AND_NK_PROGENITOR_DN	
30		REACTOME_ADAPTIVE_IMMUNE_SYSTEM	
31		YAGI_AML_FAB_MARKERS	

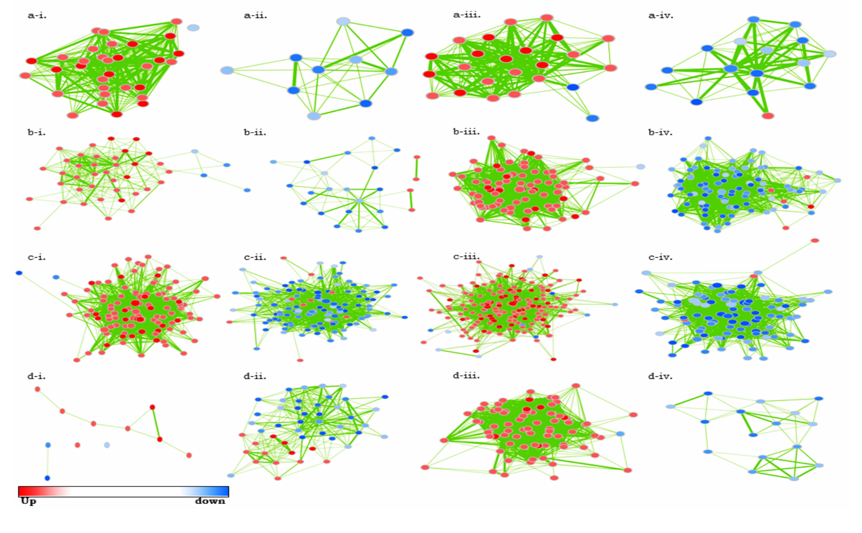

Supplementary Dataset6-Fig.3. GSEA based pathway analysis for Set2 (n=50) visualized as network (p value<0.05) for classes of (a-d) BRCA, COAD, OVCA and READ, respectively.

Supplementary Dataset6-Table3. Set2 associated Pathways in 4 classes of BRCA-COAD-OVCA-READ

NO.	Class 1	Class 2	Class 3	Class 4
		BRCA		
1	ALONSO_METASTASIS_UP	HIRSCH_CELLULAR_TRANSFORMATION_SIGNATURE_DN	KEGG_ECM_RECEPTOR_INTERACTION	ELVIDGE_HYPOXIA_BY_DMOG_UP
2	ALONSO_METASTASIS_EMT_UP	WOO_LIVER_CANCER_RECURRENCE_UP	KEGG_FOCAL_ADHESION	RHODES_CANCER_META_SIGNATURE

3	WESTON_VEGFA_TARGETS_12HR		MILI_PSEUDOPODIA_CHEMOTAXIS_DN	
4	WESTON_VEGFA_TARGETS		PID_INTEGRIN_A4B1_PATHWAY	
5	WESTON_VEGFA_TARGETS_6HR		PID_INTEGRIN1_PATHWAY	
6			CHANDRAN_METASTASIS_UP	
7			GRAESSMANN_RESPONSE_TO_MC_AND_SERUM_DEPRIVATION_UP	
**COAD**				
1	WESTON_VEGFA_TARGETS_3HR	KEGG_GLYCOSAMINOGLYCAN_BIOSYNTHESIS_HEPARAN_SULFATE	PID_AVB3_INTEGRIN_PATHWAY	REACTOME_CS_DS_DEGRADATION
2	SANA_TNF_SIGNALING_DN	REACTOME_HS_GAG_BIOSYNTHESIS	REACTOME_EXTRACELLULAR_MATRIX_ORGANIZATION	REACTOME_CHONDROITIN_SULFATE_BIOSYNTHESIS
3	RHODES_CANCER_META_SIGNATURE	REACTOME_DEGRADATION_OF_THE_EXTRACELLULAR_MATRIX	REACTOME_COLLAGEN_FORMATION	REACTOME_CHONDROITIN_SULFATE_DERMATAN_SULFATE_METABOLISM
4	REACTOME_CS_DS_DEGRADATION		KEGG_ECM_RECEPTOR_INTERACTION	WESTON_VEGFA_TARGETS_6HR
5	REACTOME_CHONDROITIN_SULFATE_BIOSYNTHESIS		REACTOME_NCAM1_INTERACTIONS	WESTON_VEGFA_TARGETS
6	REACTOME_CHONDROITIN_SULFATE_DERMATAN_SULFATE_METABOLISM		REACTOME_NCAM_SIGNALING_FOR_NEURITE_OUT_GROWTH	WESTON_VEGFA_TARGETS_3HR
7	REACTOME_A_TETRASACCHARIDE_LINKER_SEQUENCE_IS_REQUIRED_FOR_GAG_SYNTHESIS		KEGG_FOCAL_ADHESION	REACTOME_HEPARAN_SULFATE_HEPARIN_HS_GAG_METABOLISM
8	ALONSO_METASTASIS_EMT_UP		PID_INTEGRIN1_PATHWAY	REACTOME_GLYCOSAMINOGLYCAN_METABOLISM
9	ALONSO_METASTASIS_UP		KEGG_TGF_BETA_SIGNALING_PATHWAY	SANA_TNF_SIGNALING_DN
10	VERRECCHIA_RESPONSE_TO_TGFB1_C1		WOO_LIVER_CANCER_RECURRENCE_UP	VERRECCHIA_RESPONSE_TO_TGFB1_C5
11				REACTOME_CELL_CELL_COMMUNICATION
12				REACTOME_CELL_JUNCTION_ORGANIZATION
13				WEIGEL_OXIDATIVE_STRESS_BY_TBH_AND_H2O2
**OVCA**				
1	PID_INTEGRIN1_PATHWAY	CLASPER_LYMPHATIC_VESSELS_DURING_METASTASIS_DN	SUNG_METASTASIS_STROMA_UP	POOLA_INVASIVE_BREAST_CANCER_UP
2	VERRECCHIA_DELAYED_RESPONSE_TO_TGFB1	JECHLINGER_EPITHELIAL_TO_MESENCHYMAL_TRANSITION_UP	LU_TUMOR_VASCULATURE_UP	PID_INTEGRIN1_PATHWAY
3	KARAKAS_TGFB1_SIGNALING	WANG_TUMOR_INVASIVENESS_DN	REACTOME_COLLAGEN_FORMATION	VERRECCHIA_DELAYED_RESPONSE_TO_TGFB1
4	WANG_TUMOR_INVASIVENESS_UP	WESTON_VEGFA_TARGETS_12HR	PID_SYNDECAN_1_PATHWAY	KEGG_ECM_RECEPTOR_INTERACTION
5	VERRECCHIA_RESPONSE_TO_TGFB1_C5	REACTOME_PLATELET_ADHESION_TO_EXPOSED_COLLAGEN	PID_INTEGRIN1_PATHWAY	ZHANG_RESPONSE_TO_IKK_INHIBITOR_AND_TNF_UP
6	WESTON_VEGFA_TARGETS	BIDUS_METASTASIS_DN	VERRECCHIA_EARLY_RESPONSE_TO_TGFB1	KEGG_FOCAL_ADHESION
7	WESTON_VEGFA_TARGETS_6HR	SCHUETZ_BREAST_CANCER_DUCTAL_INVASIVE_UP	VERRECCHIA_RESPONSE_TO_TGFB1_C2	CHANDRAN_METASTASIS_UP
8	KEGG_ECM_RECEPTOR_INTERACTION	PID_INTEGRIN1_PATHWAY	REACTOME_EXTRACELLULAR_MATRIX_ORGANIZATION	VERRECCHIA_EARLY_RESPONSE_TO_TGFB1
9	PID_SYNDECAN_4_PATHWAY	WESTON_VEGFA_TARGETS_6HR	KEGG_ECM_RECEPTOR_INTERACTION	VERRECCHIA_RESPONSE_TO_TGFB1_C5
10	PID_INTEGRIN3_PATHWAY	SHI_SPARC_TARGETS_DN	PID_INTEGRIN3_PATHWAY	REACTOME_SIGNALING_BY_PDGF
11	GILDEA_METASTASIS	ONDER_CDH1_TARGETS_2_UP	PID_AVB3_INTEGRIN_PATHWAY	KARAKAS_TGFB1_SIGNALING
12	REACTOME_CS_DS_DEGRADATION	REACTOME_SIGNALING_BY_PDGF	REACTOME_SIGNALING_BY_PDGF	REACTOME_EXTRACELLULAR_MATRIX_ORGANIZATION
13	REACTOME_CHONDROITIN_SULFATE_BIOSYNTHESIS	WESTON_VEGFA_TARGETS	REACTOME_NCAM1_INTERACTIONS	VERRECCHIA_RESPONSE_TO_TGFB1_C2
14		REACTOME_NCAM1_INTERACT	MISHRA_CARCINOMA_ASSOCIAT	PID_INTEGRIN3_PATHWAY

		IONS	ED_FIBROBLAST_UP	
15		PID_INTEGRIN3_PATHWAY	KEGG_FOCAL_ADHESION	MISHRA_CARCINOMA_ASSOCIATED_FIBROBLAST_UP
16		AMIT_EGF_RESPONSE_60_MCF10A	CLASPER_LYMPHATIC_VESSELS_DURING_METASTASIS_DN	PID_SYNDECAN_1_PATHWAY
17		AMIT_EGF_RESPONSE_60_HELA	CHANDRAN_METASTASIS_UP	WESTON_VEGFA_TARGETS_6HR
18			RAMASWAMY_METASTASIS_UP	PID_SYNDECAN_4_PATHWAY
19			ROY_WOUND_BLOOD_VESSEL_UP	WANG_TUMOR_INVASIVENESS_UP
20			QI_HYPOXIA	LU_TUMOR_VASCULATURE_UP
21			LIAO_METASTASIS	
22			WESTON_VEGFA_TARGETS	
23			ONDER_CDH1_TARGETS_2_UP	
24			ZHANG_RESPONSE_TO_IKK_INHIBITOR_AND_TNF_UP	
25			QI_HYPOXIA_TARGETS_OF_HIF1A_AND_FOXA2	
26			WANG_TUMOR_INVASIVENESS_UP	
27			WESTON_VEGFA_TARGETS_6HR	
28			ODONNELL_METASTASIS_DN	
29			REACTOME_BIOLOGICAL_OXIDATIONS	
**READ**				
1	MISHRA_CARCINOMA_ASSOCIATED_FIBROBLAST_UP	REACTOME_HS_GAG_BIOSYNTHESIS	REACTOME_CELL_SURFACE_INTERACTIONS_AT_THE_VASCULAR_WALL	SANA_TNF_SIGNALING_DN
2		PLASARI_TGFB1_TARGETS_10HR_DN	KEGG_ECM_RECEPTOR_INTERACTION	REACTOME_CHONDROITIN_SULFATE_DERMATAN_SULFATE_METABOLISM
3		SANA_TNF_SIGNALING_UP	WANG_TUMOR_INVASIVENESS_UP	REACTOME_A_TETRASACCHARIDE_LINKER_SEQUENCE_IS_REQUIRED_FOR_GAG_SYNTHESIS
4		REACTOME_EXTRACELLULAR_MATRIX_ORGANIZATION	PID_INTEGRIN1_PATHWAY	REACTOME_CS_DS_DEGRADATION
5			LIAO_METASTASIS	REACTOME_CHONDROITIN_SULFATE_BIOSYNTHESIS
6			KEGG_FOCAL_ADHESION	
7			PID_INTEGRIN3_PATHWAY	
8			KEGG_TGF_BETA_SIGNALING_PATHWAY	
9			REACTOME_INTEGRIN_CELL_SURFACE_INTERACTIONS	
10			VERRECCHIA_EARLY_RESPONSE_TO_TGFB1	
11			QI_HYPOXIA	
12			CHANDRAN_METASTASIS_UP	
13			REACTOME_SIGNALING_BY_PDGF	

Supplementary Dataset7. Risk assessment of genes

The different classes resolved with 44 classifiers were subjected to risk assessment for all Set1, Set1-s and Set2genes using the survival analysis tool in BRB, which assigns proportional hazard ratio to each gene. Cox proportional hazards model and Wald Statistic test were used followed by calculation of hazard ratio for a two-fold change in normalized gene expression levels. Supplementary Dataset7-Table1 lists genes having significant correlation between expression pattern and survival of patient; classifiers that were thus predicted as risk genes are highlighted in red text.

Further, Kaplan Meier survival analysis plots were generated for each individual risk gene (p<= 0.01 and hazard ratio>1) followed by log rank test. Thus, 23 risk genes were identified in BRCA, 18 in COAD, 12 in OVCA, 39 in READ and 37 in GBM patients (Supplementary Dataset7-Figs. 1-5 respectively; Supplementary Dataset7-Table1). At this defined threshold, only two genes were found to be significant for LUAD cancer patients, however by relaxing stringency (p< 0.05 and hazard ratio>1) 26 risk genes were identified for LUAD (Supplementary Dataset7-Fig.6; Supplementary Dataset7-Table1).

Supplementary Dataset7-Table1. Risk assessment of genes – red highlights indicate classifiers

No.	Genes	Log rank test p value	Hazard Ratio	SD of log intensities	Sr.No.	Genes	Log rank test p value	Hazard Ratio	SD of log intensities
**BRCA (p value ≤ 0.01)**					**OVCA (p value ≤ 0.01)**				
1	ITGA11	0	1.214	1.502	1	TNFSF4	0	1.212	1.276
2	WISP1	0	1.115	1.349	2	SGIP1	0.001	1.292	0.838
3	SPON2	0.002	1.248	1.352	3	SPON2	0.002	1.146	1.348
4	NOX4	0.002	1.248	1.43	4	CTSK	0.004	1.088	1.844
5	COL5A2	0.002	1.172	1.482	5	RNASE6	0.006	1.103	1.319
6	COL3A1	0.002	1.242	0.88	6	COL3A1	0.008	1.117	1.445
7	INHBA	0.002	1.152	1.362	7	SPOCD1	0.01	1.287	0.827
8	CTSK	0.002	1.145	1.219	8	COL11A1	0.01	1.065	2.873
9	SGIP1	0.003	1.275	1.048	9	THBS2	0.01	1.087	2.033
10	FAP	0.003	1.159	1.523	10	RGS1	0.01	1.09	1.822
11	ADAM12	0.003	1.148	1.388	11	DIO2	0.01	1.089	1.309
12	MMP11	0.003	1.155	1.21	12	COL10A1	0.01	1.05	2.079
13	F2R	0.004	1.37	1.006	**READ (p value ≤ 0.01)**				
14	COL12A1	0.005	1.188	1.511	1	CLC	0	1.399	2.187
15	P4HA3	0.005	1.193	1.242	2	CTSK	0	1.581	0.932
16	PPEF1	0.007	1.205	1.383	3	NCF2	0	1.325	1.091
17	THBS2	0.007	1.14	1.656	4	CTHRC1	0.001	1.303	1.535
18	CHSY-2	0.008	1.195	1.23	5	COL6A3	0.002	1.963	0.772
19	COL5A1	0.009	1.155	1.35	6	RAB42	0.002	1.632	0.931
20	WNT2	0.009	1.104	1.669	7	COL12A1	0.002	1.448	1.072
21	CTHRC1	0.01	1.152	1.674	8	IL7R	0.002	1.188	0.952
22	PPAPDC1A	0.01	1.118	1.93	9	HLA-DPB1	0.003	1.462	1.019
23	VCAN	0.01	1.118	1.481	10	CLEC4A	0.003	1.285	1.106
**COAD (p value ≤ 0.01)**					11	WISP1	0.003	1.326	0.873
1	IL7R	0.005	1.278	1.105	12	CLEC12A	0.003	1.449	0.626
2	CLC	0.007	1.304	2.257	13	TLR1	0.003	1.216	1.095
3	PTPN22	0.008	1.353	0.713	14	F2R	0.004	1.926	0.646
4	SAMSN1	0.009	1.365	1.176	15	SAMSN1	0.004	1.277	1.084
5	CD86	0.01	1.549	1.085	16	ARHGAP15	0.004	1.321	0.802
6	VNN2	0.01	1.252	1.761	17	PLEK	0.005	1.273	1.121
7	PLEK	0.01	1.353	1.228	18	ITGB2	0.005	1.354	0.831
8	CLEC4A	0.01	1.377	1.165	19	LAPTM5	0.005	1.272	0.924
9	EDG6	0.01	1.465	1.046	20	CYBB	0.005	1.278	0.908
10	LCP2	0.01	1.399	1.049	21	CD53	0.006	1.3	1.175
11	CD53	0.01	1.302	1.295	22	RGS18	0.006	1.272	1.187
12	LILRB1	0.01	1.393	1.012	23	FCGR2B	0.006	1.216	1.475
13	ICOS	0.01	1.405	0.913	24	PTPRC	0.006	1.269	0.993
14	PSCD4	0.01	1.459	0.772	25	PSTPIP1	0.006	1.383	0.722
15	IL4I1	0.01	1.326	0.95	26	CD33	0.007	1.484	0.922
16	DOCK8	0.01	1.383	0.76	27	ITGA4	0.008	1.802	0.86
17	HLA-DPB1	0.01	1.185	1.153	28	AIF1	0.01	1.36	1.024
18	ITK	0.01	1.17	0.689	29	EVI2A	0.01	1.257	1.278

GBM (p value ≤ 0.01)					30	SH2D1A	0.01	1.414	1.022
1	MNDA	0	1.164	1.588	31	CD48	0.01	1.252	1.424
2	SIGLEC5	0	1.267	0.998	32	LY86	0.01	1.335	0.953
3	RGS18	0.001	1.198	1.474	33	POSTN	0.01	1.235	1.556
4	LAIR1	0.001	1.205	1.255	34	LCP2	0.01	1.303	0.939
5	ARHGAP30	0.001	1.258	1.005	35	CD86	0.01	1.282	1.084
6	HLA-DQB1	0.001	1.138	1.003	36	CD72	0.01	1.361	0.924
7	VNN2	0.002	1.172	1.416	37	CD52	0.01	1.231	1.311
8	CLEC4A	0.002	1.187	0.937	38	SHOX2	0.01	1.441	0.613
9	APOBEC3G	0.003	1.258	0.974	39	CDH11	0.01	1.531	1.035
10	PTPRC	0.003	1.183	1.16	LUAD (p value < 0.05)				
11	AIF1	0.003	1.133	1.446	1	SUCNR1	0.008	2.025	1.426
12	CD33	0.003	1.159	1.177	2	FCGR3A	0.01	2.215	0.847
13	EVI2B	0.003	1.078	1.105	3	LY86	0.02	1.915	1.006
14	PLEK	0.004	1.227	1.181	4	HLA-DRB5	0.02	1.769	0.912
15	TLR1	0.004	1.182	1.192	5	ARHGAP30	0.02	1.366	0.825
16	LCP2	0.004	1.221	0.927	6	FCGR2B	0.03	2.154	1.155
17	ITGB2	0.004	1.185	1.055	7	CD33	0.03	2.039	1.002
18	PTAFR	0.004	1.179	1.058	8	CD86	0.03	2.357	0.841
19	ARHGAP9	0.005	1.215	0.996	9	NCF2	0.03	1.875	0.966
20	HAVCR2	0.005	1.182	1.136	10	RNASE6	0.03	2.006	0.957
21	NCF2	0.006	1.209	1.135	11	LPXN	0.03	1.971	0.872
22	PSCDBP	0.006	1.123	1.103	12	AIF1	0.03	1.717	1.117
23	SIGLEC7	0.007	1.194	0.913	13	FCGR1A	0.03	1.836	0.975
24	CD53	0.007	1.127	1.331	14	BTK	0.03	1.82	0.874
25	CYBB	0.007	1.135	1.204	15	ARHGAP9	0.03	1.75	0.895
26	CD37	0.007	1.126	1.028	16	MNDA	0.03	1.423	1.365
27	LAPTM5	0.008	1.146	1.173	17	HAVCR2	0.03	1.704	0.924
28	LILRB1	0.009	1.232	1.093	18	CYBB	0.03	1.451	1.161
29	IFI30	0.009	1.135	1.293	19	LCP2	0.03	1.666	0.927
30	FCGR2B	0.01	1.142	1.71	20	LAPTM5	0.03	1.537	1.033
31	EBI2	0.01	1.144	1.522	21	PTPRC	0.03	1.643	0.931
32	NPL	0.01	1.197	0.974	22	NCKAP1L	0.03	1.577	0.871
33	APOBEC3A	0.01	1.22	0.865	23	FYB	0.03	1.552	0.981
34	NCKAP1L	0.01	1.157	1.027	24	CD53	0.03	1.47	1.025
35	HLA-DRB5	0.01	1.111	1.156	25	PLEK	0.03	1.464	1.011
36	BTK	0.01	1.118	1.067	26	CD37	0.03	1.383	0.867
37	CD69	0.01	1.059	1.516	-	-	-	-	-

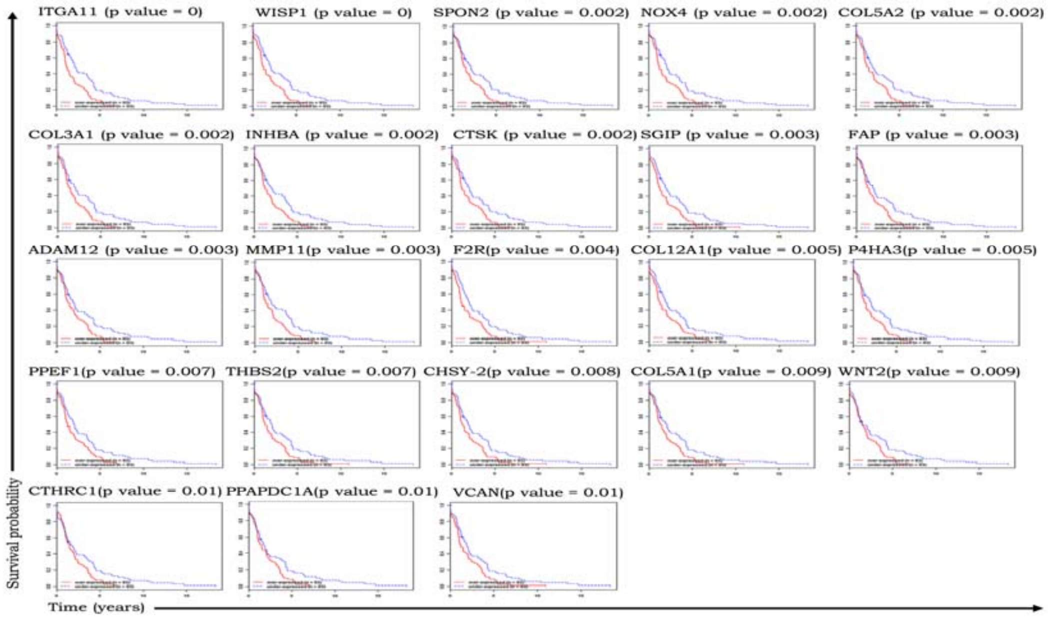

Supplementary Dataset7-Fig.1. Identification of risk genes correlating with survival of BRCA patients; Red lines indicate patients with high gene expression while blue lines indicate patients with low gene expression.

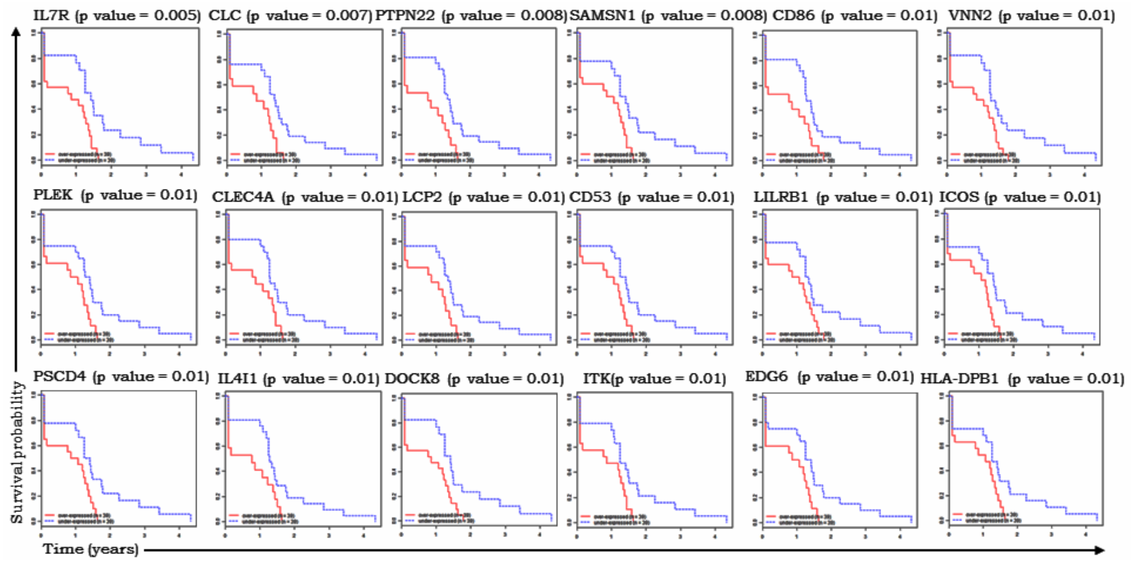

Supplementary Dataset7-Fig.2. Identification of risk genes correlating with survival of COAD patients; Red lines indicate patients with high gene expression while blue lines indicate patients with low gene expression.

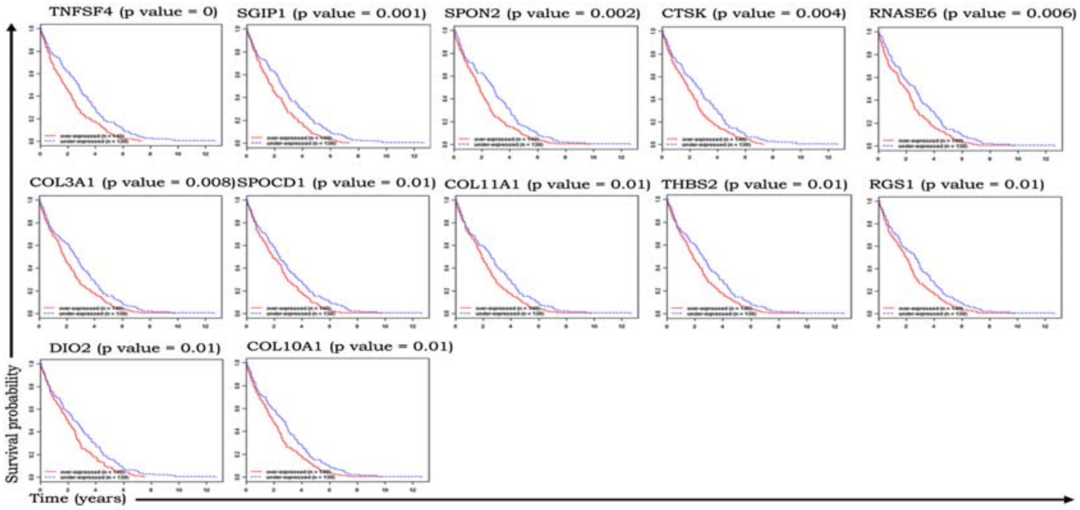

Supplementary Dataset7-Fig.3. Identification of risk genes correlating with survival of OVCA patients; Red lines indicate patients with high gene expression while blue lines indicate patients with low gene expression.

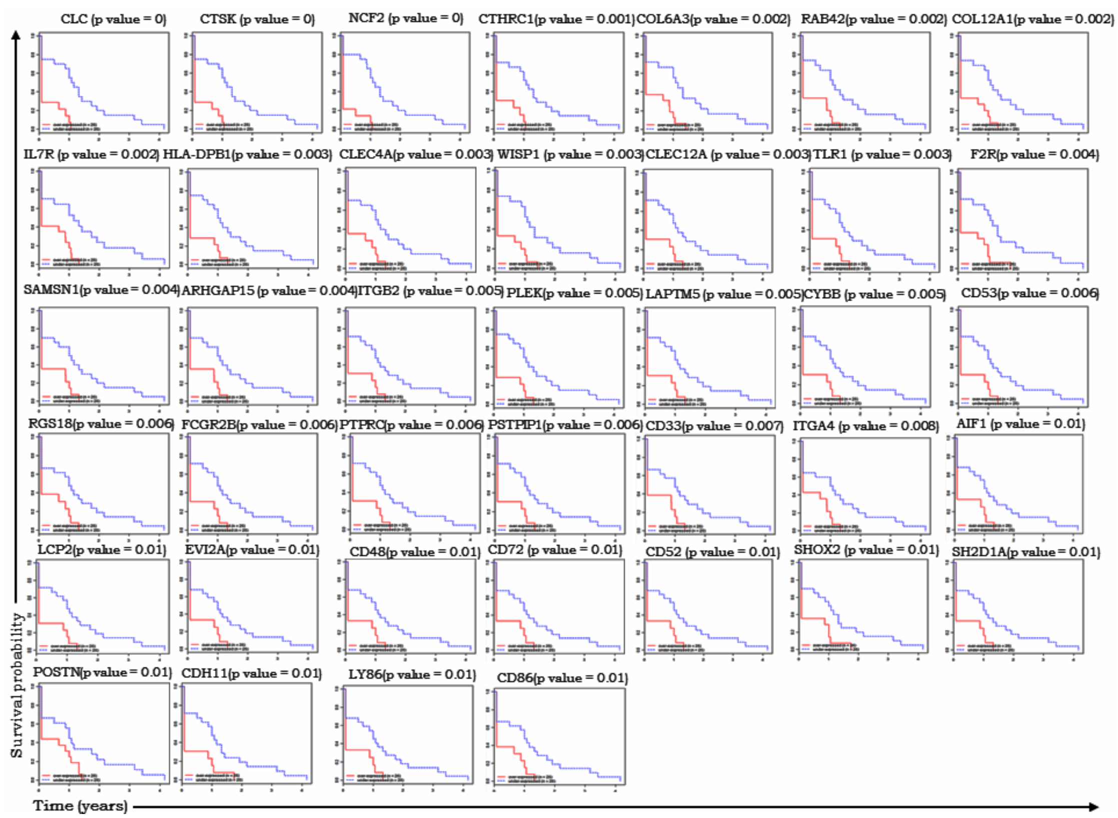

Supplementary Dataset7-Fig.4. Identification of risk genes correlating with survival of READ patients; Red lines indicate patients with high gene expression while blue lines indicate patients with low gene expression.

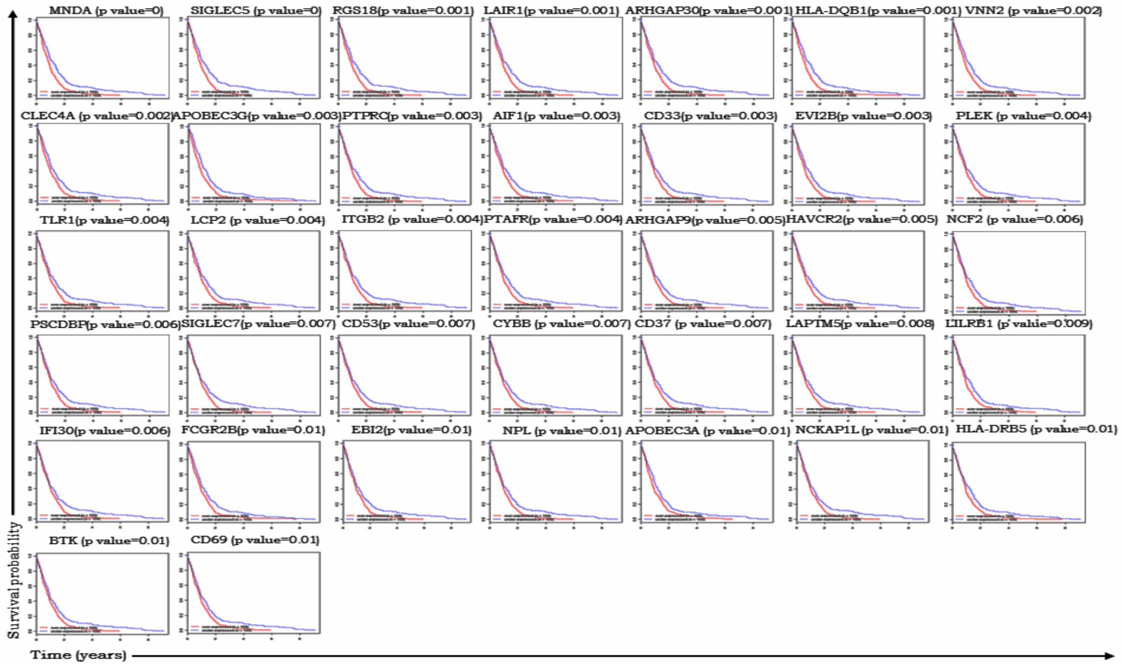

Supplementary Dataset6-Fig.5. Identification of risk genes correlating with survival of GBM patients; Red lines indicate patients with high gene expression while blue lines indicate patients with low gene expression.

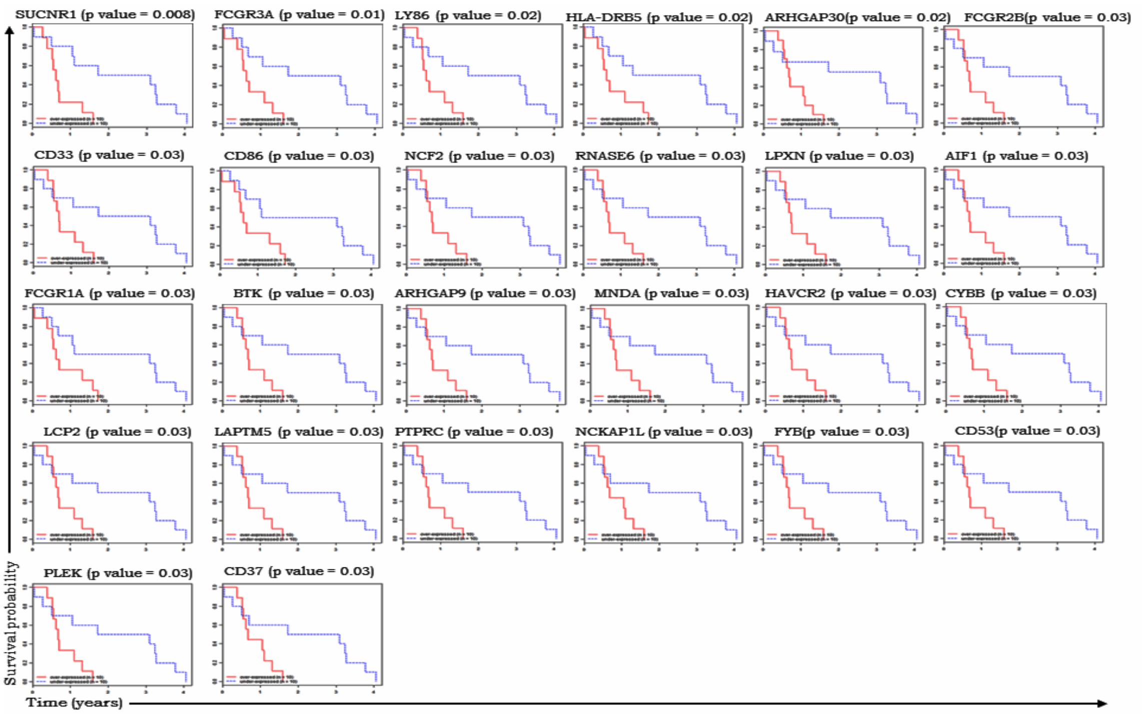

Supplementary Dataset7-Fig.6. Identification of risk genes correlating with survival of LUAD patients; Red lines indicate patients with high gene expression while blue lines indicate patients with low gene expression.

Principal Component Analysis (PCA)

Expression of 6 common risk genes for COAD-READ-GBM-LUAD (*PLEK,LCP2,CD53,MNDA, NCF2,CYBB*;p<0.05;Fig.4a-i) and 3 common risk genes for BRCA-OVCA-READ (*WISP1, CTSK, ADAM12*;p<0.05;Fig.4b-i) cancer patients were applied in PCA using Matlab software to identify association of risk genes across patients with different cancers. Weights of first three Principal Components (PCs) were as enlisted in Supplementary Dataset7-Table2.

Supplementary Dataset7-Table2. Weights of risk genes for first three PCs.

COAD-READ-GBM-LUAD			
**Genes**	**Weights in PC1**	**Weights in PC2**	**Weights in PC3**
PLEK	0.4018	-0.1402	0.1445
LCP2	0.3225	0.0845	0.0366
CD53	0.4294	0.401	-0.3261
MNDA	0.5042	-0.7767	-0.088
NCF2	0.3779	0.3043	0.7973
CYBB	0.3913	0.3414	-0.4774
**BRCA-OVCA-READ**			
**Genes**	**Weights in PC1**	**Weights in PC2**	**Weights in PC3**
WISP1	0.508	-0.5378	0.6729
CTSK	0.6437	0.7561	0.1183
ADAM12	0.5724	-0.373	-0.7302

Evaluation of sensitivity and specificity

(i) Individual risk genes of six cancers- Computation of sensitivity and specificity for each cancer type carried out for predicted high and low risk patients using individual risk genes

(p<0.05); K-M analysis for identified risk groups indicated that, expression pattern of individual risk genes can significantly predict patients with poor and better survival within six cancers (Supplementary Dataset7-Fig.7). Actual high and low risk patients were identified from TCGA data (Details are available Methods). Sensitivity and specificity of risk genes were computed for each cancer types and Mcnemar's test was used for statistical significance (Supplementary Dataset7-Table3).

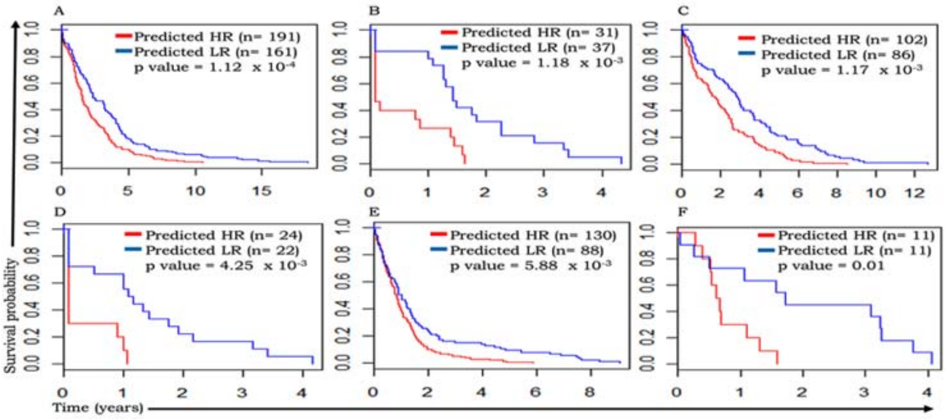

Supplementary Dataset7-Fig.7. Survival analysis of predicted risk groups based on differential expression of individual risk genes for (A-F) BRCA, COAD, OVCA, READ, GBM and LUAD, respectively.

Supplementary Dataset7-Table3. Calculation of sensitivity and specificity using individual risk genes.

Cancer types	Number of Genes	Actual HR	Actual LR	Predicted HR	Predicted LR	Sensitivity (%)	Specificity (%)	p value
COAD	39	35	34	31	37	78.57142857	83.33333333	1
READ	62	25	13	24	22	66.66666667	90.90909091	0.3
GBM	46	482	72	130	97	64.24581006	61.76470588	1.21×10^{-8}
LUAD	26	11	19	9	8	62.5	100	0.2482
BRCA	38	290	142	192	164	62.76595745	58.65384615	0.01445
OVCA	28	348	205	103	86	62.29508197	61.29032258	0.01207

(ii). Common risk genes for cancers-Differential expression pattern of 6 and 3 common risk genes for COAD-READ-GBM-LUAD and BRCA-OVCA-READ groups, respectively (Fig.4) were used for assignment of low and high risk to the patients. Sensitivity and specificity were calculated for common risk genes to predict patients with low or high risk for cancers of respective groups. READ cancer patient's risk groups were predicted by both 6 and 3 common risk genes. Common risk genes can significantly (p<0.05) predict high and low risk for GBM, BRCA and OVCA whereas sensitivities and specificities of COAD, READ and LUAD was not significant (Supplementary Dataset7-Table4).

Supplementary Dataset7-Table4. Calculation of sensitivity and specificity using common risk genes.

Cancer types	Genes	Actual HR	Actual LR	Predicted HR	Predicted LR	Sensitivity (%)	Specificity (%)	p value
**COAD**	PLEK, LCP2, CD53, MNDA, NCF2, CYBB	36	35	36	81	57.14285714	80	0.1456
**READ**		25	13	22	22	60	90	0.3711
**GBM**		482	72	127	91	66.07142857	65.71428571	1.17×10^{-7}
**LUAD**		11	19	10	10	66.66666667	77.77777778	1
**BRCA**	WISP1, CTSK, ADAM12	290	142	191	171	59.09090909	55.78947368	1.282×10^{-3}
**OVCA**		348	205	167	125	61.65803109	51.11111111	7.593×10^{-3}
**READ**		25	13	21	21	61.53846154	90	0.2207

(iii). Common and most significant risk genes-To enhance prognostic efficacy for COAD, READ and LUAD we combined common risk genes from both sets and/or individual risk genes. READ cancer patient's risk defined by common 6 (Set1) and 3 (Set2) risk genes and COAD and LUAD cancer patients were defined by using 6 common risk genes and most significant 3 genes from individual risk genes. These 15 genes were referred as prognostic signature panel indicated that, expression pattern can significantly (p<0.05) predict risk of COAD, READ, GBM, BRCA and OVCA cancer (Supplementary Dataset7-Table5).

Supplementary Dataset7-Table5. Calculation of sensitivity and specificity using common risk genes and most significant genes for COAD, LUAD highlighted in red color.

Cancer types	Genes	Actual HR	Actual LR	Predicted HR	Predicted LR	Sensitivity (%)	Specificity (%)	p value
**COAD**	PLEK, LCP2, CD53, MNDA, NCF2, CYBB, IL7R, CLC, PTPN22	36	35	56	83	58.82352941	88	0.01529
**READ**	PLEK, LCP2, CD53, MNDA, NCF2, CYBB, WISP1, ADAM12, CTSK	25	13	29	27	55.55555556	88.88888889	0.0455
**GBM**	PLEK, LCP2, CD53, MNDA, NCF2, CYBB	482	72	127	91	66.07142857	65.71428571	$1.17x10^{-7}$
**LUAD**	PLEK, LCP2, CD53, MNDA, NCF2, CYBB, SUCNR1, FCGR3A, LY86	11	19	13	14	87.5	73.33333333	0.2207
**BRCA**	WISP1, ADAM12, CTSK	290	142	191	171	59.09090909	55.78947368	$1.282x10^{-3}$
**OVCA**		348	205	167	125	61.65803109	51.11111111	$7.59x10^{-3}$

Supplementary Dataset8. Risk assessment of GBOCRL- IIPr panel through re-sampling

Random sample selection in the six cancers (from TCGA dataset - Supplementary Dataset1-Table1) was carried out using R software to generate 100 random datasets (RDs) each for BRCA, OVCA and GBM (200 samples), COAD (120 samples), READ (50 samples) and LUAD (20 samples) as described in methods and references 46,48 of manuscript. Differential expression pattern of risk genes (GBOCRL- IIPr panel; Fig.6i) were used for assignment of low and high risk of the patients. K-M analysis performed with log rank test indicated that differential expression pattern of risk genes significantly correlated with prediction of low and high risk groups within the RDs (p value <0.05; Supplementary Dataset8-Table1). A weaker correlation of the 9 genes with low and high risk groups was obtained with LUAD resampling (51%) probably due to a very limited number of available samples in the dataset (n=20).

Supplementary Dataset8-Table1. Statistical significance for survival difference in predicted low and high risk group of RDs for six cancers

BRCA (69% ; p < 0.05)		COAD (93% ; p < 0.05)		OVCA (81% ; p < 0.05)		READ (100% ; p < 0.05)		GBM (99% ; p < 0.05)		LUAD (51% ; p < 0.05)	
Random Datasets	p value	Random Datasets	p value	Random Datasets	p value	Random Datasets	p value	Random Datasets	p value	Random Datasets	p value
RD1	0.0489	RD1	0.00738	RD1	0.303	RD1	0.0049	RD1	0.00858	RD1	0.0273
RD2	0.0535	RD2	0.00070	RD2	0.0246	RD2	0.0012	RD2	0.000514	RD2	0.0854
RD3	0.0893	RD3	0.0122	RD3	0.0757	RD3	0.0035	RD3	0.00109	RD3	0.00719
RD4	0.00059	RD4	0.0126	RD4	0.0015	RD4	0.0007	RD4	0.000952	RD4	0.0815
RD5	0.0164	RD5	0.00107	RD5	0.0405	RD5	0.0023	RD5	0.00139	RD5	0.00181
RD6	0.0401	RD6	0.0627	RD6	0.0398	RD6	0.0042	RD6	0.00365	RD6	0.0503
RD7	0.00595	RD7	0.00238	RD7	0.0006	RD7	0.0023	RD7	0.0012	RD7	0.184
RD8	0.0324	RD8	0.0168	RD8	0.013	RD8	0.0019	RD8	0.000349	RD8	0.0658
RD9	0.00741	RD9	0.0329	RD9	0.0028	RD9	0.0007	RD9	0.00306	RD9	0.0488
RD10	0.00977	RD10	0.0204	RD10	0.0066	RD10	0.0004	RD10	0.000666	RD10	0.0116
RD11	0.0223	RD11	0.0096	RD11	0.0023	RD11	0.0022	RD11	0.00148	RD11	0.0383
RD12	0.0251	RD12	0.00241	RD12	0.0147	RD12	0.0042	RD12	0.000314	RD12	0.0174
RD13	0.00144	RD13	0.0128	RD13	0.0126	RD13	0.0023	RD13	0.00241	RD13	0.162
RD14	0.055	RD14	0.0158	RD14	0.0163	RD14	0.0042	RD14	0.000525	RD14	0.00193
RD15	0.0656	RD15	0.0475	RD15	0.0193	RD15	0.0018	RD15	0.000997	RD15	0.147
RD16	0.314	RD16	0.0149	RD16	0.077	RD16	0.0015	RD16	0.0514	RD16	0.00304
RD17	0.0257	RD17	0.0214	RD17	0.0201	RD17	0.0018	RD17	0.0265	RD17	0.139
RD18	0.0278	RD18	0.0634	RD18	0.0226	RD18	0.0294	RD18	0.000838	RD18	0.0258
RD19	0.00969	RD19	0.00736	RD19	0.129	RD19	0.002	RD19	0.000134	RD19	0.0509
RD20	0.0036	RD20	0.0175	RD20	0.0241	RD20	0.0040	RD20	0.00313	RD20	0.173
RD21	0.00914	RD21	0.0221	RD21	0.124	RD21	0.0055	RD21	0.00295	RD21	0.0298
RD22	0.461	RD22	0.0015	RD22	0.0136	RD22	0.0026	RD22	0.00144	RD22	0.0854
RD23	0.0126	RD23	0.0145	RD23	0.0309	RD23	0.0025	RD23	0.00016	RD23	0.0656
RD24	0.0423	RD24	0.0307	RD24	0.0436	RD24	0.0124	RD24	0.00176	RD24	0.112
RD25	0.00527	RD25	0.00515	RD25	0.0685	RD25	0.0020	RD25	0.00026	RD25	0.301
RD26	0.152	RD26	0.00164	RD26	0.0091	RD26	0.0067	RD26	9.32E-05	RD26	0.0229
RD27	0.00030	RD27	0.0787	RD27	0.0249	RD27	0.0002	RD27	0.00063	RD27	0.0654
RD28	0.00897	RD28	0.0103	RD28	0.0148	RD28	0.018	RD28	0.000298	RD28	0.0554
RD29	0.00309	RD29	0.00539	RD29	0.0137	RD29	0.0037	RD29	0.00123	RD29	0.0652
RD30	0.00138	RD30	0.022	RD30	0.0194	RD30	0.0054	RD30	0.000988	RD30	0.0554
RD31	0.217	RD31	0.0273	RD31	0.0487	RD31	0.0042	RD31	5.05E-05	RD31	0.00121
RD32	0.00787	RD32	0.00816	RD32	0.0072	RD32	0.0089	RD32	0.000231	RD32	0.00858
RD33	0.0126	RD33	0.0209	RD33	0.0228	RD33	0.0007	RD33	0.00144	RD33	0.0794
RD34	0.0221	RD34	0.00465	RD34	0.0099	RD34	0.0013	RD34	0.00137	RD34	0.0368
RD35	0.122	RD35	0.0108	RD35	0.0011	RD35	0.0062	RD35	0.00235	RD35	0.00856
RD36	0.099	RD36	0.00314	RD36	0.0791	RD36	0.0132	RD36	0.00106	RD36	0.0935
RD37	0.152	RD37	0.0189	RD37	0.0362	RD37	0.0027	RD37	0.00068	RD37	0.147
RD38	0.0326	RD38	0.0146	RD38	0.0061	RD38	0.0065	RD38	0.00781	RD38	0.0282

RD39	0.0526	RD39	0.00822	RD39	0.0304	RD39	0.0021	RD39	0.000173	RD39	0.149
RD40	0.186	RD40	0.0524	RD40	0.0263	RD40	0.0019	RD40	0.00073	RD40	0.26
RD41	0.0105	RD41	0.0139	RD41	0.0427	RD41	0.0148	RD41	0.00412	RD41	0.0742
RD42	0.00244	RD42	0.04	RD42	0.0155	RD42	0.0007	RD42	0.00168	RD42	0.0837
RD43	0.0472	RD43	0.00748	RD43	0.0258	RD43	0.0004	RD43	0.00181	RD43	0.0787
RD44	0.0113	RD44	0.00345	RD44	0.233	RD44	0.0031	RD44	0.000207	RD44	0.116
RD45	0.0128	RD45	0.00093	RD45	0.0194	RD45	0.0052	RD45	0.000893	RD45	0.0347
RD46	0.0407	RD46	0.0269	RD46	0.0161	RD46	0.0134	RD46	0.00128	RD46	0.0448
RD47	0.107	RD47	0.0318	RD47	0.0145	RD47	0.0054	RD47	0.00109	RD47	0.00793
RD48	0.0115	RD48	0.053	RD48	0.0462	RD48	0.0041	RD48	0.001	RD48	0.149
RD49	0.299	RD49	0.00227	RD49	0.156	RD49	0.0002	RD49	0.00035	RD49	0.0487
RD50	0.0386	RD50	0.00335	RD50	0.0019	RD50	0.0025	RD50	0.000955	RD50	0.0121
RD51	0.0219	RD51	0.00273	RD51	0.0265	RD51	0.0066	RD51	0.00063	RD51	0.00181
RD52	0.00081	RD52	0.013	RD52	0.0591	RD52	0.0009	RD52	0.00363	RD52	0.0091
RD53	0.466	RD53	0.0286	RD53	0.0718	RD53	0.0022	RD53	0.000374	RD53	0.0136
RD54	0.0477	RD54	0.00263	RD54	0.0039	RD54	0.0042	RD54	0.00479	RD54	0.0285
RD55	0.0257	RD55	0.00196	RD55	0.0001	RD55	0.0012	RD55	0.00167	RD55	0.0368
RD56	0.037	RD56	0.0115	RD56	0.0289	RD56	0.0004	RD56	0.00554	RD56	0.0199
RD57	0.0619	RD57	0.014	RD57	0.0864	RD57	0.0053	RD57	0.00174	RD57	0.0489
RD58	7.7x10^{-5}	RD58	0.00717	RD58	0.0005	RD58	0.0009	RD58	0.00317	RD58	0.173
RD59	0.0207	RD59	0.00122	RD59	0.0066	RD59	0.0072	RD59	0.00043	RD59	0.0163
RD60	0.0193	RD60	0.00388	RD60	0.0255	RD60	0.0022	RD60	0.00576	RD60	0.0231
RD61	0.125	RD61	0.0272	RD61	0.308	RD61	0.0022	RD61	0.00159	RD61	0.00428
RD62	0.00912	RD62	0.018	RD62	0.0089	RD62	0.0015	RD62	0.00281	RD62	0.074
RD63	0.162	RD63	0.0068	RD63	0.0961	RD63	0.01	RD63	0.000417	RD63	0.228
RD64	0.0195	RD64	0.00214	RD64	0.0064	RD64	0.0128	RD64	0.000216	RD64	0.0398
RD65	0.0017	RD65	0.0086	RD65	0.0058	RD65	0.0072	RD65	0.00569	RD65	0.0338
RD66	0.095	RD66	0.0116	RD66	0.0091	RD66	0.0012	RD66	0.00552	RD66	0.0114
RD67	0.0193	RD67	0.00388	RD67	0.0273	RD67	0.0027	RD67	0.000123	RD67	0.014
RD68	0.0145	RD68	0.00701	RD68	0.0022	RD68	0.0019	RD68	0.000233	RD68	0.017
RD69	0.0534	RD69	0.00036	RD69	0.0534	RD69	0.0176	RD69	0.0108	RD69	0.0273
RD70	0.00709	RD70	0.0691	RD70	0.033	RD70	0.0006	RD70	5.16E-05	RD70	0.0831
RD71	0.258	RD71	0.0315	RD71	0.0258	RD71	0.0011	RD71	0.000983	RD71	0.0583
RD72	0.00365	RD72	0.00234	RD72	0.068	RD72	0.0027	RD72	9.04E-05	RD72	0.09
RD73	0.147	RD73	0.00070	RD73	0.0089	RD73	0.0033	RD73	0.00135	RD73	0.0501
RD74	0.00462	RD74	0.00849	RD74	0.0432	RD74	0.0035	RD74	0.00102	RD74	0.0865
RD75	0.0244	RD75	0.00787	RD75	0.0266	RD75	0.0042	RD75	0.00527	RD75	0.0581
RD76	0.0251	RD76	0.00907	RD76	0.0060	RD76	0.0006	RD76	0.000342	RD76	0.00963
RD77	0.0219	RD77	0.00663	RD77	0.0041	RD77	0.0012	RD77	0.00084	RD77	0.0398
RD78	0.0318	RD78	0.00403	RD78	0.0184	RD78	0.021	RD78	0.000249	RD78	0.0646
RD79	0.163	RD79	0.0142	RD79	0.102	RD79	0.0076	RD79	0.00226	RD79	0.0324
RD80	0.0174	RD80	0.00122	RD80	0.0163	RD80	0.0044	RD80	0.00419	RD80	0.0921
RD81	0.00195	RD81	0.0326	RD81	0.0033	RD81	0.0108	RD81	0.00161	RD81	0.0217
RD82	0.0607	RD82	0.00862	RD82	0.0001	RD82	0.0084	RD82	0.00029	RD82	0.244
RD83	0.0128	RD83	0.00719	RD83	0.0206	RD83	0.0022	RD83	0.00116	RD83	0.0253
RD84	0.0267	RD84	0.00204	RD84	0.0379	RD84	0.0068	RD84	0.0167	RD84	0.108
RD85	0.0338	RD85	0.0106	RD85	0.0293	RD85	0.0011	RD85	0.0056	RD85	0.0837
RD86	0.054	RD86	0.00083	RD86	0.0121	RD86	0.0019	RD86	0.000446	RD86	0.122
RD87	0.0229	RD87	0.00087	RD87	0.234	RD87	0.0012	RD87	0.000428	RD87	0.0351
RD88	0.0158	RD88	0.0346	RD88	0.0547	RD88	0.0028	RD88	0.000138	RD88	0.0774
RD89	0.116	RD89	0.00251	RD89	0.0152	RD89	0.0067	RD89	0.000104	RD89	0.122
RD90	0.0552	RD90	0.0151	RD90	0.0197	RD90	0.0094	RD90	8.2 x10^{-5}	RD90	0.0488
RD91	0.0008	RD91	0.0161	RD91	0.0002	RD91	0.0034	RD91	8.5x10^{-6}	RD91	0.0854
RD92	0.00098	RD92	0.0205	RD92	0.0048	RD92	0.0034	RD92	0.0181	RD92	0.0553
RD93	0.947	RD93	0.00303	RD93	0.033	RD93	0.0018	RD93	0.0008	RD93	0.00563
RD94	0.0155	RD94	0.017	RD94	0.0098	RD94	0.0020	RD94	0.0019	RD94	0.133
RD95	0.00825	RD95	0.00136	RD95	0.0062	RD95	0.0002	RD95	0.0005	RD95	0.00998
RD96	0.0622	RD96	0.0607	RD96	0.0124	RD96	0.018	RD96	0.0050	RD96	0.00568
RD97	0.00209	RD97	0.0147	RD97	0.0017	RD97	0.0029	RD97	0.0051	RD97	0.0393
RD98	0.0289	RD98	0.0259	RD98	0.0202	RD98	0.0051	RD98	0.0106	RD98	0.0174
RD99	0.197	RD99	0.00433	RD99	0.0053	RD99	0.0003	RD99	0.0014	RD99	0.0192
RD100	0.00804	RD100	0.0488	RD100	0.0463	RD100	0.0035	RD100	0.0019	RD100	0.0583
